# Supplementary material for: A single-cell atlas of the bobtail squid visual and nervous system highlights molecular principles of convergent evolution
Source: Nat Ecol Evol. 2025 Jun 6;9(7):1245–62. doi: 10.1038/s41559-025-02720-9 (PMC12240821; doi:10.1038/s41559-025-02720-9)
Supplement: Supplementary file 1 — Supplementary Note 1. Neurotransmitter usage in cephalopod optic lobes. Supplementary Note 2. Comparison with other single-cell transcriptomic studies in cephalopods (neurons). Supplementary Note 3. Comparison of single-cell transcriptomic studies in cephalopods (glia and non-neuronal cell populations). [file 41559_2025_2720_MOESM1_ESM.pdf]

nature ecology & evolution

Supplementary information

https://doi.org/10.1038/s41559-025-02720-9

A single-cell atlas of the bobtail squid visual and nervous system highlights molecular principles of convergent evolution

In the format provided by the authors and unedited

Supplementary Discussion Note 1

Neurotransmitter usage in cephalopod optic lobes

Acetylcholine usage in cephalopod optic lobes

Acetylcholine has been isolated from the 'cerebral ganglia' of octopods[1–3] and of decapods[3–5], as well as specifically from optic lobes of octopods[6–9] and of decapods[10,11]. Presynaptic markers 'choline acetyltransferase' or ChAT (formerly known as 'Choline acetylase'), vesicular acetylcholine transporter, Vacht and choline transporter have also been investigated. In octopods, ChAT expression has been observed in the medulla of the optic lobes by [9,12,13] in Octopus vulgaris, and by ref.[14] and ref. [15] in Octopus bimaculoides. In addition to expression in the medulla, [9] and [12] observed staining in the plexiform layer of Octopus vulgaris, whereas ref. [14] observed expression in the medulla and inner granule layer but not in the plexiform layer. Vacht would be expected to co-localise with ChAT. In line with this prediction, Vacht has indeed also been observed in the medulla of Octopus vulgaris[Citation error] and in the medulla of Octopus bimaculoides [14,15]. These studies also observed expression of Vacht in the inner granule layer, where both studies also observed ChAT. In decapods, ChAT has been isolated and synthesis of acetylcholine has been observed in whole brain, 'head ganglia' or connected retina and optic lobe explants (in Loligo pealei[11,16,17]; in Sepia officinalis[18–20]; in Loligo opalescens[21]; in Lollinguneula brevis[17]; in Loligo vulgaris[22]; in unnamed squid species[23–26]. In ref. [27], the authors employed a chemical assay to determine the location within the optic lobe and observed acetylcholine (and therefore presence of ChAT) in all layers of the optic lobe cortex and medulla in Sepia officinalis.

Choline uptake has not been investigated in octapod optic lobes and only in decapod optic lobes, where uptake of choline was successfully observed, however the precise cellular location of choline transporters has not been identified (in *Loligo forbesi*[28]; in *Loligo pealei*[28–30]; in *Sepia officinalis*[19,20]).

Our stainings for ChAT (Clat, EB53255) and Vacht (EB53534) proved unsuccessful. We were however able to carry out HCR for two markers that co-localised with ChAT and Vacht in our single cell datasets: Nkx21 (EB17694) and Aces-2 (an AchE paralog, EB12863). In line with observations of Feldberg et al. [27], we observed strong staining in the medulla, faint staining in the plexiform layer and some cells in the outer granule layer. Similarly, we observed Aces-2 in cells of the medulla and the outer granule layer and in some fibres extending between the plexiform layer and outer granule layer.

Acetylcholine esterase or AchE, indeed is known to be expressed presynaptically, post-synaptically or expressed in glial cells in vertebrates and *Drosophila*. We identified three acetylcholine esterase paralogs in *Euprymna berryi*. Aces-2 was co-expressed with cholinergic presynaptic markers and Aces-3 was expressed in glial cells. Studies in other cephalopods have distinguished between butyrylcholine esterase and acetylcholine esterase, however to our knowledge, studies in cephalopods have not tried to distinguish between AchE paralogs in cephalopods. Refs. [6] and [31] observed the presence of an AchE in bulk extractions from octopod optic lobes. Ref. [32] found AchE in all layers of the cortex and in medulla fibres of *Eledone moschata*, and in the medulla, the outer granule layer, plexiform layer of the cortex but not in the inner granule layer of *Octpopus vulgaris*. Ref. [33] however

found AchE in the plexiform layer and not in the medulla. In decapods, AchE has been extracted from 'head ganglia' and 'optic ganglia' or optic lobes in bulk preps or membrane preps (from *Loligo pealei*[4,5,11,31,34]; from *Sepia offinalis*[18,31,32,35,36]; from *Todarodes pacificus* formerly known as *Ommastrephes slonaei-pacificus*[37–40]; from *Loligo opalescens*[41]). The cellular localisation of AchE in decapods was investigated in refs. [32], [42] and [43], and all three observed AchE in the medulla. [32] also observed presence of AchE in the outer granule layer, plexiform layer as well as in the medulla but not in the inner granule layer of *Sepia officinalis* optic lobes.The presence of AchE in the neuropil of medulla of *Todarodes pacificus* was only observed in [42]*. Ref.* [43] did not observe AchE in the inner and outer granule layers of the *Todarodes pacificus* cortex, but only in the r2 and r3 layers of the plexiform layer and in the neuropil of the medulla. Our Aces-2 HCR results (mentioned above - in the cells of the medulla and outer granule layer - are in line with the observations of [32] in *Sepia officinalis*. Interestingly, our HCR stainings with glial marker Eaa1-2 demonstrated signal in the plexiform layer and in parts of the outer granule layer. Although there is no sequenced publicly available genome for *Todares pacificus*, it is tempting to speculate that if *T. pacificus* as well carry several paralogs of AchE, and that ref. [43] may have observed signal from a second paralog of AchE present also expressed in glial cells present in the outer granule layer.

Metazoans are known to have two broad categories of receptors to acetylcholine: ionotropic 'nicotinic' receptors and metabotropic 'muscarinic' receptors, based on their vertebrate homologs sensitivity to these substances.

Cholinergic receptors have been identified in octopod genomes [14,44]. Intravenous administration of acetylcholine and its analogs by ref. [45] demonstrated physiological changes thought to involve the optic lobe functioning. This study included acetylcholine and agonists of ionotropic nicotinic receptors: nicotine and D-tubocurarine. Electrophysiological assays performed in ref. [46] however did not show a blockage of response after treatment with nicotinic agonist alpha-bungarotoxin in the plexiform layer, but did demonstrate and augmented pre- and post- synaptic field potentials in the plexiform layer in response to treatment with D-tubocurarine. In ref. [46], they also did not observe a blockage of synaptic response after treatment with metabotropic muscarinic acetylcholine receptor inhibitor atropine in the plexiform layer, indicating that nicotinic receptors are present in the plexiform layer of *Octopus vulgaris*. The cellular localisation of 10 nicotinic acetylcholine receptor subunits has been reported in ref. [14] in all areas of the *Octopus bimaculoides* optic lobes. One subunit oAchR H2 was not expressed in the optic lobe cortex, and two subunits oAchR G and oAchR Q were absent from the plexiform layer. In decapods, nicotinic receptors have been isolated from bulk optic lobe preparations (from *Loligo opalescens*[41]; from *Berryteuthis magister*[47–49]. In ref. [50], they performed binding assays on *Loligo pealei* and demonstrated presence of nicotinic acetylcholine receptors bound by alpha-bungarotoxin and methylcaconitine. Intravenous administration of acetylcholine and its analogs similarly to experiments in octapods revealed physiological changes thought to involve optic lobes[45]. In ref. [51], they performed electrophysiological recordings and observed that application of the nicotinic antagonist mecamylamine hydrochloride (MCM) resulted in blockade of excitatory inputs in centrifugal neurons in the outer granule layer but that application of cholinergic antagonists, both muscarinic - such as atropine and scopolamine, and nicotinic, including

MCM and dihydro-beta-erythroidine hydrobromide had no effect on either the amplitude or frequency of sEPSCs in the medulla. The only attempt to localise acetylcholine receptors in decapods was carried out in ref. [52], where the authors observed an absence of muscarinic receptors in all layers of the optic lobe of *Sepia officinalis* and presence of nicotinic receptors in the plexiform layer and medulla. We have not performed HCR stainings against any of the acetylcholine receptors or subunits as specific probe design is hindered by the expansion of ionotropic nicotinic acetylcholine receptor subunits in spiralians. Our single cell data however shows that acetylcholine receptors and subunits are expressed in the majority of cell clusters of the adult optic lobe (see Fig. 4f).

Glutamate and excitatory amino acid neurotransmitter usage in cephalopod optic lobes

Excitatory amino acid neurotransmitters glutamate (and possibly aspartate, alanine) have been examined in cephalopods. As these molecules are amino acids and are also necessary for protein synthesis their localisation has not been investigated using IHC or similar methods. Bulk quantification of free glutamate, aspartate and alanine has been carried out on octopods (in *Eledone cirrhosa*[53]; in *Octopus vulgaris*[54,55] and on decapods (in *Sepia esculenta*[56,57]; in *Sepia officinalis*[54,55]). Mitochondrial glutaminase (the enzyme that synthesises glutamate from glutamine) has not been identified in cephalopods. In our study we show that the enzyme is missing not only in the *Eurpymna berryi* genome but also in *Octopus bimaculoides, Doryteuthis pealei* and *Euprymna scolopes* genomes. We have not found any studies claiming to identify this enzyme in any cephalopod studies. Instead, the vesicular glutamate transporter Vglu has been employed as a pre-synaptic glutamatergic marker. Both refs. [15] and [Citation error] reported Vglu in the granule layers of the cortex and in the medulla but not in the plexiform layer of *Octopus bimaculoides* and *Octopus vulgaris* respectively.

Glutamate, glutamine and alanine uptake and therefore the presence of transporter molecules has also been demonstrated in optic lobes of the octopod *Eledone cirrhosa*[53] and in the decapod *Loligo pealei*[30]. Glutamatergic transporters may be situated in postsynaptic and glial cells. Similarly, the enzyme catalysing the reaction of inactivating glutamate by creating glutamine, glutamine synthase, in our study was found to be a marker of glial cells. It has also been described independently as a glial marker by ref. [15] and [Citation error] (please see Supplementary Discussion Note S3 on glia, below). Previous studies in octopods, on *Eledone cirrhosa*[53] and *Octopus vulgaris* [58] or in decapod *Rossia pacifica* [58] confirmed the presence of this enzyme in optic lobes without determining which cells expressed it.

Our study has demonstrated the presence in the *Euprymna berryi* genome of several ionotropic receptors : NMDA-type receptors, AMPA-type receptors, Kainate-type receptors, Phi-type or Phi-like-type receptors, Delta-type receptors, Lambda-type receptors. We also identified several metabotropic ‘muscarinic’ acetylcholine receptors. The presence of glutamatergic receptors in the optic lobes of cephalopods has previously been demonstrated. In ref. [45] , they administered intravenous glutamate to *Octopus vulgaris* and observed physiological effects that the optic lobe, and in ref. [46], they observed electrophysiological signal after glutamate treatment in the plexiform layer of the same species. In ref. [59], NMDAR2A/B receptors in the medulla of *Octopus vulgaris* was localised using

immunohistochemistry. In ref. [14], the presence of NMDAR1A/2A expression in the outer and inner granule layers of the cortex and in the medulla of *Octopus bimaculoides* was reported, but not in the plexiform layer. The localisation of kainate-type receptors in octopods was only investigated in ref. [14] where expression in all layers of the cortex and medulla of *Octopus bimaculoides* was observed. AMPAR-type receptors were observed by immunohistochemistry in the plexiform layer only of *Octopus vulgaris*[46]. Glutamatergic receptors have been identified in decapods as well. An ionotropic glutamate receptor dubbed sqGluR was cloned from *Loligo opalescens* and *Loligo vulgaris* [60]. AMPA-like receptors were observed through western blotting in *Loligo vulgaris* preparations of whole optic lobes[61]. In ref. [59], NMDAR2A/B was localised within the optic lobe by immunohistochemistry in *Sepia officinalis*, in the plexiform layer (r1,r3, r4), inner granule layer (in cells close to the medulla), in the medulla very few cells but many immunopositive fibres in the neuropil and an absence of signal in the outer granule layer. AMPA-like receptors were only found in the medulla[62]. In refs. [63,64], exposure of *Sepia officinalis* optic lobes to glutamate or NMDA provoked an increase in cGMP and NOS production and that this effect was countered by NOS inhibitor L-NA NMDA agonist D-AP5.

Nitric oxide in cephalopod optic lobes

Nitric oxide has been investigated in decapods outside the study by [64], linking glutamatergic NMDAR-based signalling with NO synthesis in the central nervous system of cephalpoods. There has been conflicting evidence with respect to the localisation of NADPH-diaphorase which was found to be absent in whole optic lobe of *Sepia officinalis* by [65] and [66], and also absent from the optic lobes of *Rossia pacifica* [66], while [67] observed expression in all layers of *Loligo bleekeri*. Similarly, nitric oxide synthase or NOS in *Sepia officinalis* has been reported to either be present in whole brain extracts [63,68], or present in only some layers. [69] and [70] both observed signal in the inner granule layer and in the medulla, while [69] also observed signal in the neuropil of the plexiform layer. Neither study observed the presence of NOS in the outer granule layer. We identified nitric oxide synthase in *Euprymna berryi,* (EB36054, Nos1), which was expressed in the white body clusters across samples.

Monoamines in cephalopod optic lobes

Catecholamines (dopamine, norepinephrine, tyramine and octopamine) and Serotonin synthesis, degradation and transport relies on a set of enzymes that are in some cases necessary for the synthesis of more than one neurotransmitter. The synthesis of dopamine is a two-step process requiring first the hydroxylation of the amino acid tyrosine to L-DOPA by the enzyme Tyrosine hydroxylase and in a second time the decarboxylation of DOPA to generate dopamine by Dopa decarboxylase or Aromatic-L-amino-acid decarboxylase. Norepinephrine is produced by the hydroxylation of dopamine by Dopamine beta hydroxylase (also known as tyramine beta-hydroxylase). Norepinephrine (noradrenaline) can be further methylated by Pheynethanolamine N-methyltransferase to yield epinephrine (adrenaline). Tyrosine can also be decarboxylated to yield Tyramine using Tyrosine decarboxylase. Hydroyxylation of tyramine using Tyramine beta hydroxylase (also called dopamine beta

hydroxylase - the same enzyme employed to generate norepinephrine) yields the neurotransmitter Octopamine. The synthesis of Serotonin relies on Tryptophan hydroxylase to yield 5-HTP which is further decarboxylated using Dopa decarboxylase or Aromatic-L-amino-acid decarboxylase (the same enzyme that decarboxylates L-DOPA to yield Dopamine) to yield 5-hydroxytryptamine or serotonin. Monoamine loading into presynaptic vesicles relies on vesicular monoamine transporters of Vmats that are known to load both serotonin and dopamine in vertebrates. Additionally a dopamine-specific transporter has been described in vertebrates (dopamine transporter Dat). Monoamine degradation relies on monoamine oxidase (MAO) and catechol-O-methyltransferase (COMT).

Early studies relied on fluorescence imaging techniques using which monoamine expression could be observed in octopods[71–73] and decapods[72,73], however these methods did not permit the distinction between the different catecholamines (dopamine, noradrenaline, adrenaline, tyramine, octopamine) and serotonin. In octopods, monoaminergic fluorescence was found to be absent from the granule layers but to be present in the plexiform layer and in the medulla a of the optic lobes of *Eledone cirrhosa*[72] and *Octopus vulgaris*[71,72] as well as in the synaptic plexus of the retina and plexiform layer[73]. Similarly to octopods signal was observed in the plexiform layer and medulla of the optic lobes of *Sepia officinalis*[72,73], *Alloteuthis forbesii*[73] and *Sepiola atlantica*[72].

Catecholamines and serotonin in cephalopod optic lobes : Dopamine, Norepinephrine and Epinephrine

Dopamine, noradrenaline and adrenaline has been investigated in octopods *Eledone cirrhosa*[74], *Octopus*[75], *Octopus bimaculoides*[14], *Octopus fangsiao/Octopus ocellatus*[76,77], *Octopus joubini*[17], *Octopus minor*[78] and *Octopus vulgaris*[73,74,79–81]. In octopods, dopamine has been reported in the optic lobes of *Eledone cirrhosa*[80], *Octopus vulgaris*[17,74,80,81]. Moreover, administration of resperine (an adrenergic uptake inhibitor) reduced the concentration of dopamine in the optic lobe of *Eledone cirrhosa*[80] indicative of the presence of Vmat.

Synthesis of dopamine (indicative of the presence of Tyrosine hydroxylase and Dopa decarboxylase) was observed in *Octopus joubini* and *Octopus vulgaris*[17]. Retinal perfusion with L-DOPA enhanced electroretinograms (ERGs) indicative of the expression of Dopa decarboxylase (also known as Aromatic-L-amino-acid decarboxylase) in retinal tissues. Administration of dopamine antagonist 6-hydroxydopamine into the systemic heart resulted degeneration of glial cells and neurons in the plexiform zone of the optic lobe of *Octopus vuglaris*, indicating to the presence of monoamine re-uptake transporters[82].

Moreover, stimulation of optic lobe efferents after incubation with dopamine induced screening pigment migration in the retina, and that this migration was prevented by incubation with SCH 23390 (dopaminergic antagonist) or dopamine depletion by treatment with reserpine and dark adaptation in *Octopus fangsiao/ Octopus ocellatus*[77]. Moreover,

sectioning the optic tract decreased the total levels of dopamine in the whole optic lobe of *Octopus vulgaris*[74].

Noradrenaline was demonstrated in the optic lobes of *Eledone cirrhosa*[80] and *Octopus vulgaris*[74,81]. As with dopamine, resprine treatment of *Eledone cirrhosa*[80] reduced the concentration of noradrenaline indicative of the presence of Vmat. In contrast, in ref. [17], they did not observe synthesis of noradrenaline in the optic lobes of *Octopus joubini* and *Octopus vulgaris*. Optic tract sectioning reduced the levels of noradrenaline in whole optic lobes[74]. Adrenaline was found absent from both *Eledone cirrhosa* and *Octopus vulgaris* optic lobes through chromatographic methods[80]**.**

In contrast to the monoaminergic fluorescence imaging, more recent studies revealed presence of dopamine, L-DOPA-synthesis enzyme tyrosine hydroxylase and dopamine transporter in the outer granule layer, but absent from the plexiform layer (dopamine in *Octopus minor*[83]; Tyrosine hydroxylase and dopamine transporter in *Octopus bimaculoides*[14]). These more recent studies however did concur with the studies relying on biogenic fluorescence with respect to signal in the medulla.

Inhibitory neurotransmitters : GABA, Glycine, Taurine usage in cephalopod optic lobes

GABA or γ-Aminobutyric acid is considered the main inhibitory neurotransmitter in mammalian systems. In octopods, GABA has been detected at low levels in whole optic lobes in *Octopus*[84], *Eledone cirrhosa*[53,85–88], or was found to be absent from the optic lobes of *Eledone muschata*[89] and *Octopus conispadicus*[90]. Whatsmore, ref. [89] showed that Octopus vulgaris hepatopancreas was incapable of deaminating GABA, indicating that the neurotransmitter is absent in the whole animal. Similarly, GABA uptake (and therefore a GABA transporter) was found to be present at low levels in Eledone cirrhosa[53] in *Eledone cirrhosa.* In ref. [17] , authors didn't observe evidence of GABA synthesis (and therefore of glutamate decarboxylase or GAD, the enzyme that synthesises GABA) in *Octopus joubini* and *Octopus vulgaris* eye and optic lobe connected explants, while in ref. [53], they observed low levels of GAD in *Eledone cirrhosa* optic lobes. More recent studies, however, have observed the presence of GABA through immunohistochemistry in whole *Octopus vulgaris* optic lobes[54] and in the medulla of *Eledone cirrhosa* and *Octopus eledone*[91]. In *Eledone cirrhosa* but not in *Octopus eledone*, in addition, signal was observed in the tangential layer of the plexiform layer of the cortex. In ref. [14], they cloned oGAD from *Octopus bimaculoides*, but no spatial expression pattern was described. In decapods, evidence is similarly contradictory. On one hand, in earlier studies, GABA was found to be absent from the optic lobes of *Sepia esculenta*[57], and that *Sepia officinalis* hepatopancreas could not deaminate GABA[89]. In a later study on the other hand GABA was found to be present in whole optic lobes of *Sepia officinalis* and *Loligo vulgaris*[54]. synthesis of GABA in the optic lobes of *Lolliguneula brevis* and *Loligo pealei* was not observed in ref. [17], whereas the presence of GAD was reported in all layers of the *Sepioteuthis lessoniana* optic lobe in ref. [92].

Evidence of the presence of GABA receptors in octopods comes from intravenous administration of GABA to *Octopus vulgaris* and *Eledone cirrhosa*[45], where physiological changes possibly attributable amongst other to optic lobe function were observed. In ref. [46], on the other hand, no changes in electrophysiological readings after treatment with picrotxoin (an inhibitor of GABA receptors) were observed, while in decapods, bicuculiine-sensitive GABA receptor binding was observed[93]. Intravenous delivery of GABA to *Sepia officinalis* and *Alloteuthis subulata* revealed physiological effects that may require optic lobe function[45]. Similarly microinjection of GABA into optic lobe medulla neuropil in ref. [94] induced similar physiological reactions and injection of picrotoxin (GABAA receptor agonist) produced general arousal of the animal at high concentrations. Localisation of GABA receptors in optic lobes has not been investigated in octopods or decapods to our knowledge.

The second common inhibitory neurotransmitter is glycine, which is also an amino acid and as such its nature therefore impedes the usage of immunohistochemical methods for its investigation. In octopods, glycine has been demonstrated to be present using chemical quantification assays and HPLC in *Eledone cirrhosa*[95] and *Octopus vulgaris*[54]. Uptake of glycine indicating the presence of a glycine transporter was demonstrated in *Octopus*[91]. In decapods[54] showed the presence of glycine in the optic lobes of *Sepia officinalis* and *Loligo vulgaris*, however leaving animals in the dark did not affect the concentration of glycine in optic lobes [55]. Uptake of glycine and therefore the presence of a glycine transporter was observed in *Loligo pealei* synaptosomal preparations[30]. Glycinergic receptors have not been described in octopods or decapods in the optic lobe. Intravenous administration of glycine to Octopus vulgaris did not reveal any physiological effects, as was observed in using GABA or picrotoxin in the same study[45].

Taurine or 2-aminoethanesulfonic acid is a sulfonated amino acid derivative. Its presence has been detected in the whole optic lobes of octopods *Eledone cirrhosa*[88,96] and *Octopus vulgaris*[54]. Administration of intravenous taurine to *Octopus vulgaris* in ref. [45] did not produce any physiological effects that could be attributed to optic lobe function. Similarly, taurine has been detected in the whole optic lobes of decapods *Sepia officinalis* and *Loligo vulgaris*[54]. Although taurine was detected in both the retina and optic lobe, leaving the animal in the dark did not affect the levels of taurine produced by optic lobes[55].

In our study we identify the E.berryi the taurine transporters Sc6a6-1 and Sc6a6-2 and the closely related Gadl1 and Cdo1 the enzymes that synthesise taurine, but that are also involved in GABA synthesis.

Histamine usage in cephalopod optic lobes

The molecule histamine is widely involved in many processes, including immune function in vertebrates. In octopods, histidine (the precursor to histamine) was quantitatively isolated and identified through HPLC in ref. [54]. In the optic lobes of *Eledone moschata,* the presence of histamine was moreover demonstrated[97] and those histamine levels were decreased after treatment with a monoamine oxidase inhibitor and reserpine treatment. In decapods histamine was detected in whole optic lobes of *Sepia officinalis* and *Loligo vulgaris*[54]. In ref. [98], they used in situ hybridisation to localise histidine decarboxylase, the enzyme that

synthesises histamine to the medulla only of *Sepia officinalis.* We identified a histidine decarboxylase in *Euprymna berryi* (EB01849, Dchs-2), which was not found to be expressed at meaningful levels or in more than a few cells in any of our single cell datasets.

Neuropeptides and neurohormones in cephalopod optic lobes

Neuropeptides are neuronal signalling molecules that regulate various biological processes and act as neurotransmitters, neuromodulators or neurohormones. They are thought to be an "evolutionarily ancient property of neurons"[99]. Neuropeptides are derived from larger precursor proteins that are processed through the secretory pathway to intracellular dense core vesicles until they undergo exocytosis. They typically bind G-protein-coupled receptors. Neuropeptides are traditionally classified into families. A high throughput study carried out by [100] employing *de novo* transcript assembly from RNA-seq, coupled with mass spectrometry has provided an exhaustive list of neuropeptides in the optic lobes of *Sepia officinalis*. The full list of neuropeptide ligands and receptors in *Euprymna berryi* is available in Table S8.

The achatin neuropeptide was first identified in the gastropod *Achatina fulica*[101,102]. Achatin One Achatin 1 (ALEESFKSDGAamide) and two Achatin 2 (ADANAEDYSEAILR and LLLENYGARL) have been isolated and identified from the optic lobes of *Sepia officinalis*[100]. While ref. [103], describes the isolation of Ocp-1 and Ocp-3 cardioactive peptides that may derive from the same precursor protein as Achatins in *Octopus minor* optic lobes. In our annotation of neuropeptides through sequence homology, we identified three Achatin-like ligands: putative Achatin (EB36398, Unchar-26442), putative Achatin 1 (EB46637, Unchar-33860) and putative Achatin 2 (EB48924, Unchar-35527). We also identified three putative receptors to Achatin (EB19839, EB32109, EB36398, respectively preliminarily named by Top Uniprot best blast hits as Anr, Ccapr-3 and Npy6r-2). Putative Achatin 1 ligand Unchar-33860 was found to be expressed at low levels in all single cell datasets including hemocytes. There were more cells found expressing Unchar-33860 in photoreceptors clusters. Among putative Achatin receptors, EB19839 and were expressed at low levels notably in the Glutamatergic 1, Cholinergic1, Cholinergic2 and Dopaminergic6 clusters of the mature optic lobe.

Allatostatins (ASTs) or buccalins have been identified in insects and molluscs. Ref. [100] identified two AST A1/A2 peptides (GMDPMMFGHLamide and MDPMMFGGLamide) in *Sepia officinalis* optic lobes and ref. [104] observed evidence of Buccalin in *Octopus maya*. In our gene annotation, we identified three potential allatostatin-like ligands (Allatostatin 3-like : EB49390, Unchar-35839; Allatostatin1/2-like: EB52623, Unchar-38109; Asta-C-like: EB04628, Unchar-3274). Unchar-38109 was observed to be expressed in mature optic lobe Glutamatergic3 cell cluster. Unchar-3274 was observed to be expressed in the hatchling optic lobes in clusters Glutamatergic4, Serotininergic, Glial1, Neuro1, Neuro3, Neuro4 and Neuro6. We also identified four potential receptors to Allatostatins (Asta-A-like receptor: EB47363, Ar; Asta-C-like receptor: EB02215, Drd5; Asta-C-like receptor: EB15572, Ssr2-1; Asta-C-like: EB49232, Ssr2-2). EB47363 was expressed at low levels in the mature optic lobe in Glutamatergic1 and Cholinergic1. EB49232 was expressed at low levels in a few serotoninergic cells in the hatchling optic lobe and in Glutamatergic3 in mature optic lobes.

Allostatin receptors are phylogenetically related to myoinhibitory peptide (MIP) receptors in spiralians[105]. We moreover identified two myoinhibitory peptide ligands in *Euprymna berryi* (EB48153, Atg12; EB53349, Unchar-38610) that may resemble Asta-B, that were found to be expressed in a sparse and very small number of cells in optic lobe single cell datasets.

Allatotropins are known to act in the insect nervous system. Ref. [100] identified one Allatotropin-family neuropeptide (GFKDNVSNRIAHGFamide) in the optic lobes of *Sepia officinalis*. Allatotropins have not been described in the optic lobes of octopods to our knowledge. We did not identify an allatotropin receptor but did identify an allatotropin ligand (EB26196, Unchar-18815), that we observed expression for in our single cell datasets at very low levels in very few cells in the retina and optic lobe datasets.

APGWamide tetrapeptides were first identified in the gastropod *Fusinus ferrugineus*[106] . It is known to male sexual behaviour in *Lymnaea stagnalis*[107]. The annelid RGWamide has been proposed to be orthologus to the mollusc APGWamide [108]. In cephalopods, APGWamides have been described in both octopods and decapods. In octopods, APWamide ligand was detected in the plexiform layer of *Octopus vulgaris* optic lobes[109]. Refs. [100], [110] and [111] detected APGWamide in the optic lobes of *Sepia officinalis*, while evidence of APGWamide in the optic lobes of *Idiosepius pygmaeus* was recovered[112]. In our annotation of the *Euprymna berryi* genome we did not identify any ligands or receptors of APGWamides.

ASWLDFamides have been reported in Brachiopods and Nemerteans[105]. We identified a single ligand in *Euprymna berryi* (EB37437, Unchar-27182).

Bursicons A and B have been described in arthropods and spiralians[105,113,114]. In ref. [100], the authors did not observe expression of Bursicons in the optic lobes of *Sepia officinalis*. We identified four Bursicon-like ligands in *Euprymna berryi* (Bursicon A: EB11050, Sspo-2; Bursicon B: EB18724, Unchar-13579; Bursicon A/B: EB44784, Unchar-32505; Bursicon B: EB44844, Unchar-32543). Unchar-13579 was observed to be expressed at low levels in Dopaminergic4 cluster in the mature optic lobe. Unchar-32505 was expressed in the hatchling optic lobe in Neuro5, Neuro6, Tyraminergic, Glutamatergic1 and Glutamatergic3.

Calcitonin and its calcitonin-gene related peptide (CGRPs) are considered to be neurohormones that have been detected in vertebrates, arthropods and spiralians[105]. CGRPs have been observed in the optic lobes of the octopod *Octopus vulgaris*[115] and retinas of *Octopus vulgaris*[116], the optic lobes of the decapod *Sepia officinalis*[117] and in of the nautiloid *Nautilus macromphalus*[117]. CGRP expression was absent from the outer granule layer and medulla of *Octopus vulgaris* but present in the tangential fibres of the inner quarter of the plexiform layer, in varicose fibres of the outer granule layer. In our annotation of the *Eurpymna berryi* genome, we identified a single calcitonin-like ligand (EB52826, Unchar-38253), which was not observed to be expressed in any of the cell clusters at meaningful levels in more than a handful of cells.

Luqin / Cardio-excitatory Peptides have been described in spiralians[100,105]. We identified a single Cardio-excitatory-like peptide in Euprymna berryi (EB20073, Unchar-14627), which was found to be expressed at low levels throughout the hatchling optic lobe (most prominently in Neuro4 cell cluster) but only in Dopaminergic11 in mature optic lobes. We also identified two receptors resembling Luqin/RYamide receptors (EB07494, Ryar-2 and

EB08114, Ryar-3) that were not expressed a meaningful levels or in more than a handful of cells in any of our single cell datasets.

The neurohormones Crustacean Cardioactive Peptides (CCAPs) were originally described in crustaceans and subsequently identified in gastropods[118] and described in other lophotrochozoans[105]. In ref. [100], they identified three CCAPs in the optic lobes of *Sepia officinalis* (VFCNSFGGCQN, VFCNSFGGCTNI and VFCNSYGGCKSF). In our annotation of the *Euprymna berryi* genome, we identified a single CCAP ligand (EB00267, Unchar_182), which was found to be expressed in the hatchling optic lobe cell cluster Neuro6. We did not identify any CCAP receptors in *Euprymna berryi*.

Choleocystokinin/Sulfakinins are known in vertebrates and invertebrates[100,105]. In ref. [100], they did not observe any CCKs in the *Sepia officinalis* optic lobe. We identified five putative receptors in *Euprymna berryi* for CCKs/Sulfakinins (EB01449, Cckar-1; EB32767, Cckar-10; EB32784, Cckar-11; EB17427, Cckar-4; EB32255, Cckar-9). Cckar-1 was expressed prominently in the photoreceptor clusters of the retina and in the mature optic lobe clusters Dopaminergic3, Dopaminergic5, Dopaminergic6, Dopaminergic7, Inhibitory1 and Inhibitory2. It is noteworthy that Dopaminergic5 and Dopaminergic6 as highlighted in the main text also express FMRFamide. Cckar-9 was expressed at low levels in Dopaminergic7 in mature optic lobes.

CLCCY neuropeptides were identified in annelids, brachiopods and phoronids[105,119] and have been described as lophotrochozoan-specific[120]. We identified twelve CLCCY-like ligands in *Euprymna berryi* (EB15753, Unchar-11452; EB00185, Unchar-127; EB00285, Unchar-192; EB28440, Unchar-20438; EB00470, Unchar-309; EB00535, Unchar-352; EB00603, Unchar-399; EB55910, Unchar-40468; EB00696, Unchar-461; EB07608, Unchar-5571; EB00098, Unchar-66; EB09463, Unchar-6941). We did not detect meaningful expression of any of these genes in our single cell datasets.

Clionin has been described in molluscs and coincidentally, but it was not did not detected in the optic lobes of *Sepia officinalis*[100] . We identified a single Clionin-like ligand in *Euprymna berryi* (EB15753, Unchar-23624). Unchar-23624 was observed in the hatchling optic lobe cell clusters Neuro1, Neuro3, Neuro4, Neuro5, Tyraminergic, Serotoninergic, Glutamatergic3 and Glutamatergic4).

The receptors to DH44/ Egg Laying hormone are known to be phylogenetically related [105]. We identified four DH44 receptors (EB18987, Crfr2-2; EB31284, Crfr2-3; EB31304, Crfr2-4; EB46191, Crfr2-6), none of which were expressed in a meaningful fashion on our single cell datasets.

Thiel et al. found ‘dkk_related’ peptides in phoronids, brachiopods and nemerteans[105]. We identify a single dkk_related ligand in Euprymna berryi (EB19090, Unchar-13853), that was found to be expressed at low levels in several dopaminergic clusters (Dopaminergic1, Dopaminergic4, Dopaminergic8, Dopaminergic9).

The EP/CCHamide receptor has been described for its function in swimming behaviour of nemerteans [121]. We identify two Endothelin/ GRP/ CCHamide/ EP receptors in *Euprymna berryi* (EB31751, Cch1r; EB05299, Unchar-3784). Unchar-3784 is strongly expressed in glial

cells in hatchling optic lobes and Dopaminergic6, Dopaminergic4, Cholinergic1 and Glutamatergic1 in mature optic lobes.

Ecdysis triggering hormone (ETH) has been described in Ecdysozoa performing a function necessary for moulting and subsequently found in other animals as well[105,122,123]. We identified a single ETH receptor in *Euprymna berryi* (EB19173, originally named Cckar-6 after the top hit in Uniprot). It was not found to be expressed in any of our single cell datasets.

The neuropeptide corticotropin-releasing factor has been described in the octopod *Octopus vulgaris*[124]. We are not aware of any studies investigating corticotropin-releasing factors in the optic lobes of decapods. In our annotation of the Euprymna berryi genome, we identified two potential CTRF receptors (EB18987, Crfr2-2 and EB46191, Crfr2-6), neither of which was expressed at meaningful levels in any of our single cell datasets, which is in line with the absence of reports of CTRFRs. Calcitonin/DH31 receptors have been described to (L-DCc) are known to form a consertved set of lophotrochozoan receptors[105]. We identified three DH31-like receptors in *Euprymna berryi* (EB16621, Calcr-1; EB17810, Calcr-2; EB16626, Calrl-1), neither of which were expressed at meaningful levels in or in more than a few cells.

The neuropeptide gonadotropin-releasing factor (GNRH) has been reported in bulk optic lobes of decapods *Loligo bleekeri* and *Sepia officinalis* [100,125]. We have identified a ‘progonadoliberin’ or (GNRH) gene (EB43306, Gon1) in the *Eurpymna berryi* genome. We also identified two potential GNRH receptors (EB20294, Gnrhr-1 and EB20405, Gnrhr-2). Neither the ligand nor the putative receptor gene were found to be expressed at meaningful levels in more than a few cells.

FFamides are involved in male reproduction in molluscs. [100] identified two FFamides in the optic lobes of *Sepia officinalis* (GLNPNVNSLFFamide and VYAPTRGQQNPHSYamide). In the *Euprymna berryi* genome, we identified a single FFamide ligand (EB38055, Unchar-27625) that was expressed at negligible levels in the optic lobe datasets.

The neurohormones FLGamides were first reported by ref. [100] in *Sepia officinalis*, where they identified five FLGamides (GFDSLGGGSFIGV, SFDSLGGGAFLGamide, SFDSLGGGSFLGamide, TFDSLGGGSFLGamide and TFDSLGGGSFLGamide). We did not identify any FLGamides in the *Euprymna berryi* genome.

FVRIamide neuropeptides were originally identified in gastropods[126], in which they are involved in reproductive behaviour. [100] identified seven FVRIamides in the *Sepia officinalis* optic lobes (ANAFLRIamide, GGASSFLRIamide, IPASSFVRIamide, NPLSQFVRIamide, SNPRGFLSVGSRFVRIamide, SNPSTFLRIamide and TNQFLRIamide). In our annotation of the Euprymna berryi transcriptome, we identified 11 FVRIamide-like ligands and no receptors (EB15841, Mkln1; EB14689, Unchar_10708; EB19946, Unchar_14533; EB23793, Unchar_17275; EB36180, Unchar_26278; EB37692, Unchar_27382; EB38317, Unchar_27827; EB04270, Unchar_3006; EB41611, Unchar_30153; EB08058, Unchar_5914; EB07933, Yrd6-16). Notably, Unchar-10708 was expressed in photoreceptors (Photoreceptor_r1 and _r2b) at very low levels in a few cells.

Galanin is a neuropeptide known to be expressed in the central and peripheral nervous systems and in the endocrine system of mammals[127]. Galanin was investigated in the octopod *Octopus vulgaris* by immunohistochemistry and was found to be present in the

fibres of the plexiform layer and the cell islands of the medulla[128]. In *Euprymna berryi*, we did not identify a galanin ligand but did identify two putative galanin receptors (EB26522, Galr2-1 and EB33607, Galr2-2), neither of which were expressed at meaningful levels or in more than a few cells.

The neurohormone LASVGLXamide has been described in molluscs[113]. Zatylny-Gaudin *et al.* identified seven LASGLXamides in the optic lobes of *Sepia officinialis* (AFDPLASGLIamide, ELDTLASGLIamide, PFDPLASGLIamide, PFDSLASGLIamide, pQFDHLASGLIamide, SFDPLASGLIamide and SLDPLASGLIamide)[100]. We did not identify any LASVGLXamides in *Eupymna berryi*.

The neurohormones Leucokinins (LKs) are known to be expressed in molluscs [113]. Zatylny-Gaudin *et al.* identified a single LK-like peptide SRFSPWHamide that was found to be expressed in *Sepia officinalis* optic lobes[100]. In *Euprymna berryi*, we identified three potential LK-like ligands, two Leucokinin1-like (EB27934, Mib1 and EB36346, Unchar-26407) and one resembling Leucokinin/Leucokinin2 (EB36564, Unchar-26560). Of which, EB27934 was expressed at low levels throughout several of the tissues we sampled for our single cell analysis, notably in the Photoreceptor_r1 population.

Neurohormone LFRFamides known for its effects in molluscs have been reported in whole *Sepia officinalis* optic lobe[100,129] (NSLFRFamide, SRTFFRYamide and TIRFamide). In *Sepia officinalis,* LFRFamide has been observed in the inner granule layer and medulla[130] while in *Sepiella japonica* staining has only been observed in the medulla[131]. We did not identify any LFRFamides in *Euprymna berryi*.

Myomodulin has been described in the optic lobes of octopod *Octopus maya* [104] and in decapod *Sepia officinalis*[100]. In Euprymna berryi, we identified a single myomodulin ligand (EB18578, Unchar-13482) that was not found to be expressed in any of the single cell datasets.

Neuropeptide Y and Neuropeptide Y3 have been described extensively in molluscs and vertebrates. In octopods, NPY-like peptides have been investigated in *Octopus vulgaris* optic lobes[124,132]. Suzuki *et al.* [132] observed NPY immunoreactivity in the plexiform layer in fibres and the cell islands and varicose fibres of the neuropil of the medulla, but absent from the granule layers. In decapods, Smart *et al.*[133] reported NPY-like molecule in *Loligo vulgaris* in dissected brain tissues (optic lobes, sub and supra-oesophageal ganglia) using mass spectrometry, while [100] identified four NPY peptides, two NPY1 (pQNNGGAAPQSPEELTNYLKALNEYYAIVARPRFamide, YAIVARPRFamide) and two NPY3 (APKIPGELSEYLKALSDYYAIAARPRFamide, pQEGLLAAPKIPGELSEYLKALSDY) in the optic lobes of *Sepia officinalis*. In *Euprymna berryi,* we identified four putative receptors to NPY-4 or NPY/NPF (EB03473, Fmar-2; EB11851, Npfr-2; EB11977, Npfr-3; EB11751, Npy6r-1). EB03473 was notably expressed at low levels in photoreceptor cell clusters and throughout many clusters in the mature optic lobe. We also identified an NPY ligand (EB37793, Npy-1 and EB38253, Npy-2). Npy-1 and Npy-2 were expressed in the Neuro2 cell cluster of the mature optic lobe at low levels.

Neuropeptide KYs also known as bradykinin-like neuropeptides have been described in molluscs. Zatylny-Gaudin *et al.*[100] reported three NKY peptides in the optic lobes of *Sepia officinalis*, two NKY1 peptides (PAQGYVPVPRTSNINNSDGSGSSVIRYamide and TSNINNSDGSGSSVIRYamide) and one NKY2 peptide (VPAFGSFFSPSPSDNSDTSKIFRYamide). In *Euprymna berryi*, we identified a single NKY2/myomodulin-like ligand (EB46581, Tc3a-29), which was expressed in the Neuro6 cluster of hatchling optic lobes and Bradykinin-like ligand (EB29324, Unchar-21088) which was sparsely expressed some optic lobe cells.

The neuropeptides Orcokinins were originally identified in the crayfish[134]. Orcokinins have been described in the octopod optic lobes in *Octopus maya*[104] and in "sparse, scattered population throughout the deeper region of the medulla" in *Octopus bimaculoides* [15]. In the decapod Sepia officinalis, Zatylny-Gaudin *et al.* [100] reported six Orcokinin B peptides (PFDSIDSSAFGGMamide, PFDSISDSAFGGMamide, pQFDSISHSSFRQMamide, SFDSIASSGFGGMamide, SFDSIDGGMFRTMamide and SFDSIDGSAFGGMamide). We did not identify any Orcokinins in *Eurpymna berryi*.

Pedal peptides have been described in different species. Zatylny-Gaudin *et al.* [100] reported ten pedal peptides in the optic lobes of *Sepia officinalis* (HLDYIGSGLI, NFDLIGSGLI, NFDSIGSGLI, NFDSVGSGLI, NFDTVGSGLV, NIDSIGSGLI, NLDRIGSGLV, NLDSIGAGLI, NLDSIGSGLV and NLDSVGAGLI). [135] described the cellular localisation of pedal peptide-like molecules in *Sepiella japonica*. The authors found Sj-PP to be present in outer and inner granule layers as well as in the medulla but absent from the plexiform layer. the In *Euprymna berryi*, we identified six Pedal protein-like ligands (EB22790, Unchar-16618; EB40123, Unchar-29119; EB04436, Unchar-3127; EB04789, Unchar-3392; EB05827, Unchar-4217; EB12819, Unchar-9345). Unchar-16618 was highly expressed in a number of cell clusters in hatchling optic lobes including Glutamatergic2 (but notably less highly expressed in cholinergic and GABAergic cell clusters, glial clusters and the white body cluster). In mature optic lobes Unchar-16618 was observed in Dopaminergic5 and Dopaminergic6, which notably are the two clusters that also express FMRFamide. Unchar-3127, Unchar-3392 were found to be highly expressed in the Glutamatergic3 cell cluster of the hatchling optic lobes. Unchar-4217 was expressed in some of the mature dopaminergic optic lobes cell clusters (Dopaminergic2, Dopaminergic3, Dopaminergic4, Dopaminergic7, Dopaminergic8 and Dopaminergic12).

PRQFVamides have been reported in the optic lobes of the octopod Octopus maya[104] and the decapods *Sepia officinalis*[100] and *Sepiella japonica*[136]. In *Sepiella japonica* PRQFVamide-related peptide-encoding transcript was absent from the plexiform layer but present in the granule layers and medulla. We did not uncover any PRQFVamides in *Eupymna berryi*.

PTSP-like neurohormones have been described in molluscs. Zatylny-Gaudin *et al.*[100] reported six PTSP-like peptides in the optic lobes of *Sepia officinalis* (DRPDTWNSMNTWamide, DWDSLQAWamide, NPDTWDSMSAWamide, NPNTWDSMAAWamide, SPDTWDSMSAWamide and SPNTWDSMAAWamide). In *Eupymna berryi*, we identified a single PTSP-like ligand (EB34204, Unchar-24838) that was not expressed in any of our single cell datasets.

PXXXamide neuropeptides were described in protosomes. Zatylny-Gaudin *et al.* [100] reported a single PXXXamide peptide in the optic lobes of *Sepia officinalis* (TSSDQRIAELQALIALSNTIGHGQVNPEEIamide). We did not identify any PXXXamide peptides in *Eupymna berryi*.

Somatostatin/ Urotensin peptides were originally identified in vertebrates and secondarily observed in molluscs. In the optic lobes of the octopod *Octopus minor*[83] described somatostain immunoreactivity in the outer granule layer. In decapods, somatostatin has been detected in the optic lobes of *Loligo pealei*[137,138] and in *Sepia officinalis*[100] finding two peptides (GGMGLCLWKVCPTAPWM and GGMGLCLWKVCPTAPWMRST). Immunohistochemistry in *Todarodes pacificus* (formerly known as *Ommastrephes sloanei pacificus*) demonstrated the presence of somatostatin in all layers of the optic lobe. In Euprymna berryi, we identified a single Urotensin-like ligand (EB35252, Unchar-25585) that was expressed in very few cells, though notably was observed in Neuro3 and Neuro5 cell clusters of the hatchling optic lobe.

Tachykinins are a large family of neuropeptides identified in both vertebrates and invertebrates. Zatylny-Gaudin *et al.*[100] identified seven tachykinins in the *Sepia officinalis* optic lobes (AAPFYHGFVASRamide, AHASLGFVGSRamide, FSPYAFQGSRamide, ISAEAFAPSRamide, LSSQAFFGSRamide, pQPASLGFVGSRamide and YSALGFMGSRamide). In Euprymna berryi, we identify a Tachykinin-like ligand (EB38107, Unchar-27664) and putative Tachykinin receptor (EB07570, Tlr2-1). The ligand was observed to be expressed at low levels in the Neuro2 cell clusters of the mature optic lobe, while the receptor was observed to be expressed at low levels in the Glutamatergic1, Cholinergic1 and Dopaminergic10 cell clusters of the mature optic lobe.

The oxytocin/vasopressin family of peptides are known in both vertebrates and invertebrates. Peptides from this family have been reported in the optic lobes of the decapod *Sepia officinalis*. Bardou *et al.*[139] observed vasopressin-like immunoreactivity in the plexiform layer (t2,t3 and t4) and in the fibres of zone of the radial columns and in the zone of tangential bundles of the medulla as well as in optic tract bundles and oxtyocin-like immunoreactivity in all layers of the optic lobe. Similarly, [140] reported presence of cephalotocin and octopressin using RT-PCR and mass spectrometry on the optic lobes of *Sepia officinalis*. Bardou *et al.*[141] observed oxytocin-like immureactivity in the granule layers of *Sepia officinalis* optic lobes only after 15 days and high expression in the medulla. In Euprymna berryi, we identified four vasopressin/oxytocin receptors (EB18644, Ctr1-1; EB37522, Ctr1-3; EB45546, Ctr2-3; EB18513, Opr), all of which were found to be expressed at very low levels and in very few cells.

We additionally identified two Neuroparsin ligands (EB49703, Bmper-2 and EB12280, Unchar-8956). Unchar-8956 was notably expressed at low levels in Glutamatergic3 of the mature optic lobes.

7-B2 ligand (EB53305, Unchar-38579) was expressed at low levels in photoreceptor cell cluster cells, highly expressed in all cell clusters in the hatchling optic lobes and in some of the neuronal cells of the mature optic lobe, many of the dopaminergic clusters: in Dopaminergic1, Dopaminergic4, Dopaminergic5, Dopaminergic6, Dopaminergic7, Dopaminergic8, Dopaminergic9, Dopaminergic10, Dopaminergic11; Cholinergic1; Glutamatergic1, Glutamatergic2, Glutamatergic3).

Supplementary Discussion Note S2.

Comparison with other single-cell transcriptomic studies in cephalopods (neurons)

Here we provide a detailed discussion of our findings in the context of three contemporaneous manuscripts describing single cell transcriptomics of cephalopods as they pertain to neurons.

Among neuronal cells, all four studies observe ELAV as a marker of neuronal identity. In both Songco-Casey et al. and our datasets, we observe the expression of synaptotagmin orthologs, amyloid precursor protein and tetraspanin paralogs as broad markers of neuronal identity. Songco-Casey et al. and Duruz et al. both observe an additional pan-neuronal marker: CUG triplet repeat binding protein orthologs in neuronal cells, which we observe only in two dopaminergic cell clusters (Dopaminergic1, Dopaminergic9)[15,142]. We concur with Duruz et al. on the expression in photoreceptor cells of rhabdomeric photoreceptor markers opsins, retinochrome, transient receptor potential channel, arrestin, retinal binding protein, phospholipase C, Gq alpha, but also other molecules: Poly(rC) binding protein 3, E3 ubiquitin protein ligase RNF167, N-myc downstream regulated gene, amyloid precursor protein, calsyntenin and cytochrome 5 orthologues.

Duruz et al. did not classify cell types based on neurotransmitter usage. Styfhals et al, identified dopaminergic neurons based on the expression tyrosine hydroxylase, whereas Songco-Casey employed dopamine transporter Dat. We identified both markers in our dataset. Furthermore, all three studies (our's, Styfhals et al., Songco-Casey et al.) observe a population of cells expressing presynaptic markers of both dopaminergic and glutamatergic neurons[15,142]. In our study they correspond to Dopaminergic4, Dopaminergic5, Dopaminergic6, Dopaminergic7, Dopaminergic8, Dopaminergic10, Dopaminergic11. However due to the absence of availability of differentially expressed gene lists, we can only state at this time that the only known marker in common between these studies to our knowledge was the glutamatergic presynaptic marker vesicular glutamate transporter. We observe expression of Fmrf-amide in a set of dopaminergic and glutamatergic neurons (Dopaminergic5, Dopaminergic6) and expression of Six4 in another set of glutamatergic and dopaminergic cells (Dopaminergic10, Dopaminergic11). Songco-Casey et al. also observe two sets of dopaminergic cell clusters expressing Fmrf-amide in a set of dopaminergic-only cell clusters (clusters 10-11) and another set of dopaminergic-only cell clusters expressing of Six3/4/5 (clusters 12-17), however Fmrf-amide+ or Six3/4/5+ in Songco-Casey's dataset are in dopaminergic only cells and not in glutamatergic and dopaminergic cells. Songco-Casey et al. observe dopaminergic populations expressing additional prqfv1 and 2 neuropeptides. We do not observe expression of this neuropeptide in any of our cell clusters. Songco-Casey et al. observe expression of Dscam in their fmrf1+ dopaminergic population (clusters 10-11). We observe some Dscam expression in Dopaminergic5, which is also a glutamatergic and dopaminergic cluster.

All three studies (our's, Styfhals et al., Songco-Casey et al.) observe several glutamatergic cell populations. All three studies find the vesicular glutamate transporter (Vglu2/Vglut2) as a

common marker. Songco-Casey et al. similar to us also observe in some of the glutamatergic cells, the expression of a Vat1l and sonic hedgehog orthologues.

All three studies also identified cholinergic cells. Styfhals et al. employed vesicular acetylcholine transporter Vacht as a key marker, Songco-Casey et al. employed Vacht (that they refer to as slc18a3) and choline acetyltransferase ChAT. We also observed the expression of acetylcholine esterase Aces-2 expressed in cholinergic neurons in both mature and hatchling optic lobes (Cholinergic1 and Cholinergic2 in mature optic lobes; Cholinergic2 in hatchling optic lobes). We observed expression of two additional homeobox transcription factors Otx2 and Nkx2.1 in Cholinergic1 and Cholinergic2 respectively in mature optic lobes. Songco-Casey et al. also observed separate expression of nkx2 and otx in cholinergic clusters 7 and 8+9 respectively.

Several smaller populations of neurons were not consistently identified across datasets. The neurotransmitter tyramine is synthesised using the enzyme tyramine decarboxylase (Tdc1) from tyrosine. To make the neurotransmitter octopamine a subsequent step requires the enzyme tyramine beta hydroxylase that catalyses the synthesis of octopamine. We captured a tyraminergic neuronal population of cells only in hatchling optic lobes identified by the marker tyrosine decarboxylase. Styfhals et al. as well as Songco-Casey et al. observed an octopaminergic population expressing tyramine beta hydroxylase. We did not observe expression of tyramine beta hydroxylase, whether at hatchling or mature stages. Another difference between our study and that of Styfhals et al., and Songco-Casey et al., are serotonergic cells. Using tryptophan hydroxylase 2 and the serotonin transporters Sert and Sc6a4-1, we also observed a serotoninergic population in the hatchling optic lobe and only a few cells at mature stages. Styfhals et al. also observed a serotoninergic population in *O.vulgaris* expressing tryptophan hydroxylase 2 and serotonin transporter Sert in *O.vulgaris* optic lobes. Songco-Casey et al. did not identify a serotoninergic population in *O.bimaculoides* optic lobes. Similarly, we and Styfhals et al. observed small inhibitory neuronal populations. Styfhals observed based their identification on vesicular GABA transporter vgat and glutamate decarboxylase enzyme, gad. Songco-Casey et al. point out in their manuscript that they fail to find gad expression in their dataset. Our labelling of these cell populations as inhibitory was based on a process of elimination. These clusters expressed low levels of makers known to be associated with presynaptic inhibitory neurotransmitter synthesis or transport and an absence of presynaptic markers of other neurotransmitters. We provide an exhaustive list of makers for these cell clusters. Songco-Casey et al. observed an additional population expressing the neuropeptide orcokinin (cluster 37). We did not observe expression of this neuropeptide in the optic lobe. Styfhals et al. identified additional clusters each expressing a specific neuropeptide: APWGamine, Bursicon, Fmrf1 and Fmrf3. We did not observe such populations in our datasets.

Supplementary Discussion Note 3.

Comparison of single-cell transcriptomic studies in cephalopods (glia and non-neuronal cell populations)

Here we provide a detailed discussion of our findings in the context of three contemporaneous manuscripts describing single cell transcriptomics of cephalopods as they pertain to glia and other non-neuronal cell types.

Hemocytes

Among non-neuronal populations identified in more than one study, the hemocyte population stands out (as it was observed in all four studies). We observe orthologous marker gene expression only in common with Duruz *et al.* including genes such as Ets4, actin paralogues, EF-hand domain containing D2, Gata transcription factor orthologs, neurotrypsin, Glutathione-S-Transferase, ferritin, Gelsolin, Cd63, Myosin regulatory light polypeptide and phospholipase. Styfhals *et al.* list vascular endothelial growth factor and von Willebrand factor as markers of hemocytes that we do not observe in our *E. berryi* datasets. Songco-Casey *et al.* mention acetylcholine esterase as their marker of the hemocyte population, which we also do not observe in either our hemocyte-only dataset nor our retinal or optic lobe-specific datasets[15].

Retinal cell populations

Duruz *et al.*[142] and our study are the only ones of the four to have identified a pigmented epidermal population and the lentigenic cells of the eye. Similarly, we observe several common orthologous markers to those observed by Duruz *et al.* in pigmented epithelial cells, namely the tyrosinase enzyme, Xaa-Pro aminopeptidase and the Ras-related protein Rab-32. Similarities between lentigenic cells observed in both the Duruz *et al*. whole embryonic head dataset and our retinal dataset are however limited to the expression of S-crystallins.

Glial cells

Among the most interesting non-neuronal cell populations is the glial population. With respect to glial markers we are in agreement with the two octopus studies[15,143] with regards to two markers glutamine synthetase (Glna-2 in our study) and excitatory amino acid transporter (Eaa1-2 in our study). Duruz *et al.* did not observe any glial cells in their squid study. We provide an exhaustive list of differentially expressed genes found at both the hatchling stages and mature stages, as well in two clusters of glial cells at each stage. This is to our knowledge the first such exhaustive list. We additionally find several markers present in both glial clusters at both mature and hatchling stages (transforming growth factor beta induced protein Bgh3-3 and uncharacterised proteins Unchar-9359 and Unchar-16827), some present in only some of the hatchling glial populations (Hatchling glial1 & mature optic lobes: ferritin Fris-3, janus kinase and microtubule-interacting protein 1 Rimb1, sestrin ortholog Sensn3, melanotransferrin Trfm-1, innexin protein Unc7-2 and uncharacterised proteins Unchar-7728, Unchar 29982, Unchar-10760, Unchar-35702; Hatchling glial2 & mature optic lobes: uncharacterised protein Unchar-4561; hatchling glial2 & mature optic lobe glial1:

uncharacterised protein Unchar-4561), along with genes specific to each glial cell population at each stage.

Note on glial cell definition

Glial cells were first described by Virchow in 1846 as a connective structure only ("nervenkitt") in reference to glue[144]. Glia have since been defined as non-neuronal cells involved in a number of support functions including protection of neuronal tissue against structural deformation, providing a permeability barrier, speed impulse conduction, removal and repair of neurons, involvement in guidance, differentiation and growth of neurons, regulation of ionic composition of the neuronal environment, neurotransmitter uptake, inactivation or release and metabolic interaction with neurons[145]. Additional functions have been sporadically described in some organisms, including electrical coupling through desmosomes in honeybee drone glia[146].

Regarding glia, we stress that in metazoans, many different cell types have been collectively dubbed 'glia'. These cell types are thought to be (1) non-neuronal cells associated with neurons, (2) that do not conduct fast currently and (3) do not possess neurotransmitter bearing vesicles in presynaptic structures[147]. Other authors have proposed that glial cells must also descend from neuronal precursors[148]. These criteria would however exclude many canonical peripheral vertebrate glial cells that derive from the neural crest. Altogether, these rather vague criteria however are in contradiction with the criteria proposed by Raff in order to determine whether two cells belonged to the same cell type[149]: (1) cell fate, (2) morphology and behavior, (3) time and place of origin, (4) cell lineage-restricted gene expression (5) cell lineage. The large variety of cells referred to collectively as 'glia' in different clades have different embryonic origins and carry out different sets of functions in different clades and different cell types have been observed at different stages of ontogeny (see below). For these reasons, cells carrying out these support or 'glial' functions have been proposed to have evolved convergently several times in evolution[150,151], and ***therefore we do not know whether all glial cells throughout evolution ought to express the same markers even within cephalopods.***

Below as requested by reviewers we propose an overview of glial cells in vertebrates and invertebrates. We focus initially on most investigated clades (vertebrates and arthropods for which the most information is available).

Vertebrate glia

In the nervous system of adult vertebrates, there are broadly five types of glial cells : astrocytes (and satellite cells), oligodendrocytes (and Schwann cells), ependymal cells, enteric glia and microglial cells.

Astrocytes and satellite cells maintain an appropriate chemical environment for neurons. Astrocytes derive from 'radial glia' originating from neuroepithelial cells and from the neural crest, whereas satellite cells derive from neural crest progenitors[152,153]. Multiple sub-cell types of astrocytes have been described including tanycytes and pituicytes. Interestingly, Müller glia in the vertebrate retina originate from the neural crest and from multipotent retinal progenitors[154]. Transcriptional markers of astrocytes include Eaat1, whose ortholog Eaa1-2

we observe in our glial datasets (see above). Among canonical markers of astrocytes, many do not have orthologs in the *E. berryi* genome (e.g connexin 43) or have several one-to-many orthologs that are not expressed in glial cells in our datasets. Interestingly, S100b and Glial fibrillary acidic protein (GFAP) are genes that have been frequently utilised to justify homology between glial cells across taxa. In our phylogenetic survey, we do not observe orthologs of these genes in the *E. berryi* genome (Orthogroups OG0009961 and OG0000105 respectively which are limited to vertebrates). Curiously, mouse Aquaporin 4 is considered a marker of astrocytes in mammals, has several one-to-many orthologs of which one (Aqp4-1) is expressed in adult OL Glial1 population whilst its paralog Aqp4-3 is expressed in the *E. berryi* white body.

Oligodendrocytes and their peripheral nervous system counterparts Schwann cells provide myelin sheath for axons[155], action potential propagation, neurotransmitter secretion, providing lactate to axons for aerobic ATP production and participate in functional plasticity of the adult central nervous system and behaviour[156–158]. In mammals, oligodendrocytes derive from several Oligodendrocyte progenitor cell populations within the brain[159], which themselves derive from specific radial glial populations[160]. Canonical markers of oligodendrocytes in mammals include Myelin Basic Protein, Myelin oligodendrocyte glycoprotein, Olig1, Olig2, Sox10, claudin 11. Schwann cell precursors derive from neural crest cell progenitors [161]. Canonical markers for Schwann cells include S100b, Myelin Basic Protein, Myelin Protein Zero, P75NTR, NCAM, GAP43, Sox10, Oct6/Egr2 and Krox20. Cephalopods are known to not produce or employ myelin to ensheath axons and it is therefore not surprising that we did not find orthologs of genes associated with myelin in the *E. berryi* genome. Two mouse Olig2 orthologs were found in the *E. berryi* genome (Olig2, Bhe22), however neither of these genes was found to be expressed in glial cells. Similarly, the Sox10 ortholog in *E. berryi* Sox9 was not expressed in glial cell clusters in our datasets.

Ependymal cells are ciliated epithelial cells that line the ventricles of the brain and spinal canal. These cells derive from radial glia. Their functions include regulation of cerebrospinal fluid, blood-brain-barrier formation, metabolism and homeostasis and clearing of waste from the brain[162]. Canonical mammalian markers of ependymal cells include GFAP, Vimentin, S100. Vimentin is another gene whose expression has been employed to justify potential homology between glial cells in different taxa. In our single cell dataset, vimentin is not expressed.

Enteric glia derive from a specific population of neural crest cells[163] and participate in regulating enteric reflexes and communication between neurons innervating the digestive tract. Enteric glial markers include PLP1 and S100b while GFAP is specific to a subset of cells[164]. As mentioned above we do not find orthologs of S100b or GFAP in our analysis and the sole *E. berryi* gene family member of Orthogroup OG0001535 is not expressed in glial cells in our dataset.

Microglial cells derive from erythromyeloid progenitors in the yolk sac and have been referred to as brain's resident macrophages[165–168]. More recent studies have demonstrated more nuanced roles played by microglia in removing cell debris, regulating neuronal survival and synaptic remodeling, communication with oligodendrocytes as well as playing an important role in regulating neurodegeneration[169]. Canonical microglial markers in mammals include AIF1, Pu.1, Vimentin, P2Y12, Tmem119, Cxcr1, Cd68, Hexb, Sall1. Microglial cells in

mammals are part of the immune system, a role that is carried out in cephalopods by hemocytes. It is therefore not reasonable to seek homology between *E. berryi* glial cell populations and mammalian microglia. We did not identify orthologs of these genes except for Hexokinase and Salm, neither of which were expressed in glial cells in our datasets.

Invertebrate glia

(1) Porifera

Poriferans are not known to have glial cells. However, it has been suggested that epithelial cells are able to feed through the specialisation of the apical cell domain may have constituted precursors to the glial cells[170].

(2) Cnidarians

No bone fide glial cells have been identified in cnidarians. One study has suggested that epithelial cells in *Tripedalia cystophora* may provide a glial-like function[171], while a more recent studies on the sea anemone *Nematostella vectensis* have suggested that specialised neural progenitor cells may give rise to non-neuronal support cells that could be likened to glial cells [170,172], and neuronal cells with non-typical morphology expressing *gcm* and *eaat1* could represent ‘protoglia’ that quench glutamate[173,174]. Transcriptome-wide homology between vertebrate- or *Drosophila*-like glial cells were not uncovered in high-throughput single cell transcriptomics of *N. vectensis*[175]. As mentioned in the paragraph devoted to glial cells in *Drosophila*, we identified an *E. berryi* ortholog (Gcm) of *Drosophila* gcm, however this gene was not expressed in our dataset. We did however identify Eaa1-2 (ortholog of vertebrate Eaat1) as a marker of glial cells.

(3) Acoels

Glial-like cells have suggested to be present in acoels[176,177]. The single cell transcriptomics study of acoel uncovered a potentially glial cluster expressing lipoproteins receptors ldlr1, lrp2 [178]. We did not identify orthologs of these genes in the *E. berryi* genome.

(4) Urochordates

Support cells were described in larval stages of *Oikopleura dioica* ontogny[179]. The neural tube of larval *Ciona* contains ciliated cells dubbed ‘ependymal’ in reference to the vertebrate ependymal glial cells[180], whilst adult sessile *Ciona* feature potentially morphologically glial-like cells[181], sheaths surrounding neurons[182], ambiguous or accessory cells associated with synapses[183]. Single cell transcriptomic studies have confirmed the existence of a cluster potentially corresponding to ‘ependymal cells’ (cluster 5) expressing Aquaporin 9, zeb, gonadotropin-releasing hormone b and KH.C10.493[184]. We did not identify orthologues of these genes expressed in glial clusters in our datasets.

Astrocyte-like cells expressing an ortholog of glutamine synthetase were described in the ascidian *Styela plicata*[185]. Glutamine synthetase (Glna-2 in our study) is indeed one of the markers expressed by *E. berryi* glial cells in our study (see above).

(5) Echinoderm glia

Support cells have been described in asteroids, holothuroids, ophiurids and echinoids[186]. Descriptions of unipolar and bipolar cells dubbed ‘radial glia’ in Echinoderms (due to their

resemblance to the 'radial glia' of chordates). These cells secrete glycoprotein subcomissural organ (SCO)-spondin (also known as Reissner's substance)[186]. These 'radial glia'-like cells are precursors to glia and neurons[187] and participate in neuroregeneration[188]. Echinoderm radial glia exhibit immunoreactivity to ERG1[188]. A homolog of the *Drosophila* glial marker *gcm* homolog exits in the genome of sea urchins but is not expressed in these cells. Markers of radial glia in echinoderms include Myc and Brn1/2/4 orthologs and proliferation markers as these cells are thought to be the only proliferating cells in the nervous system of animals belonging to this clade [186]. Several orthologs of *scso-spondin* were observed in the *E. berryi* genome, however, none of them were expressed in glial clusters. Similarly, an ortholog of *gcm* was identified but was not expressed in our datasets. In addition, markers of activated glial cells active in regeneration in echinoderms have been described, which include Myc and Brn1/2/4[186]. No orthologs of these genes were expressed in our glial clusters.

(6) Ecdysozoan glia

The nematode *Caenorhabditis elegans* has also been described to carry multiple sets of glial cell populations: sheath glia, socket glia, and mesodermally derived glial cells associated with peripheral sensory structures. Their functions include support, regulation of neuronal activity, promoting synaptogenesis, maintenance of synaptogenesis and regulating neuronal and engulfing of dead neuronal cells[189–191]. Among cell-type specific markers have been reported for pan-glial cell types (mir-228, delm-1 and itx-1), and specific sub-cell types (for example: amphid and phasmid sheath glia, amphid and phasmid socket glia, cephalic sheath glia, inner labial socket glia[192]). We did not identify any orthologs of these genes expressed in glial clusters in our datasets.

The most exhaustively investigated glial cells among ecdysozoans relate to *D. melanogaster.* Adult *Drosophila* glia is classified based on their location into inner and outer surface glia, cortex glia, ensheathing glia or astrocytes. Surface glia are further subdivided in perineurial and subperineurial glia, although in some cases these labels may have been misused (reviewed in [193]). Surface glia form the equivalent of the blood brain barrier in Drosophila. Cortex glia surround neuronal cell bodies in the cortex of the brain. Ensheathing glia surround neuropils or axonal tracts. Astrocytes are also located within the neuropil and interact with synapses thanks to extensive projections[194,195]. Each of these broad categories are further subdivided into sub cell types. Single cell transcriptomics studies have however revealed that glial cells previously distinguished on a morphological level could not be distinguished at the transcriptional level[195–198].

The transcription factor *glial cells missing* (*gcm)* is transiently expressed in all embryonic glia except for mesectoderm-derived glia and is considered a marker gene of glia[148,199]. We identified an ortholog of *Drosophila gcm* in the *E. berryi* genome, however this gene is not expressed in our single cell datasets. Drosophila *gcm* acts upstream of *repo* which is another pan glial marker in *Drosophila*. We did not identify a clear ortholog of Drosophila repo in the *E. berryi* genome. Extensive studies have characterised single gene markers for glial sub-cell types including lncRNA *CR34335, CG6126, Indy, Wun2, Gs2, Gat, Eaat1, alrm, hoe1, wrapper*. Except for *Eaat1* and *Gs2* whose *E. berryi* orthologs Eaa1-2 and Glna-2 are indeed a marker of glial cells in our study, we did not detect expression of orthologs of any of the single gene marker in our *E. berryi* glial clusters.

We took advantage of the recently published series of single cell transcriptomics studies investigating the *Drosophila* nervous system and eye[197,198,200–202] to compare expression of genes expressed in *Drosophila* larval and adult glial cells and did not identify any transcriptome-wide homology between neuronal or glial cells in our datasets and each *Drosophila* dataset [data not shown].

Glial-like cells have also been described based on morphology in other members of Panarthropoda including the horseshoe crab *Limulus polyphemus* (five glial cell types named based on morphological similarity to vertebrate glial cells: peripheral glia, 'Schwann cells', stellate, vellate and vascular neuroglia)[203], the lobster *Homarus vulgaris*[204], antarctic isopod *Glyptonotus antarcticus*[205], as well as in onychophrans [206]

(7) Lophotrochozoan glia

Morphological descriptions of glial-like cells in bryozoa have been reported in adult and larval stages[207,208] (and disputed by others[151,209]), in platyhelminthes where glial-like cells have been proposed to have evolved convergently at least five times[210] (in cells of derived parasitic cestode platyhelminths, vertebrate immunoreactivity to vertebrate glial marker S100b was observed[211]), in gastrotrichs[212,213], in phoronids have been reported in adult and larval stages [214,215], in brachiopods[216], nemertea[217].

Motile cells resembling microglia have been described in the medicinal leech *Hirudo medicinalis* containing exosomes containing RNA[218,219], and astrocyte-like cells were described to regulate calcium and potassium concentrations necessary for neuronal excitation[220–223].The cell lineage of these cells is unknown.

In the annelids morphological examinations have identified glial-like cells in *Armandia brevis*, *Protodrilus* and *Nereis*[224–226]. *Mytilus* and *Nereis* putative glial cells were shown to express IL1-alpha gene ortholog and to animals exhibited chemotaxis to IL1-alpha[227]. Radial glia expressing intermediate filaments have been reported in *Owena fusiformis* and *Magelonidae* [228,229] and morphological descriptions of glial-like sheath cells were reported in a study of 22 species of annelids in mushroom body-like structures[230]. Morphological descriptions of support cells dubbed glial cells were proposed in Sipuncula[231].

We focus on descriptions of glia in cephalopods in the following section. In this paragraph, we cover information regarding glial in non-cephalopod molluscs. Multiple types of glial-like cells have been described in molluscs. In *M. stultorum*, such cells participate in neurotransmitter uptake and inactivation[232]. In the mussel *Mytilus edulis*, two cell types were found to regulate osmoregulation [227]. Potential glia cells were described to express vertebrate glial cell markers GFAP, intermediate filament and vimentin orthologs were reported in *Megalobulimus abbreviatus*[233]. In the snail *Helix pomatia,* support cells modulating calcium regulation have been described[234]. The putative glial cells of snail *Planorbis* were reported to store glycogen for use by adjacent neurons[235]. *Aplysia* glia were first reported to express secretory molecule Ag [236], while *Lymnaea* glial cells expressed AchBP, which was subsequently confirmed to be expressed in *Aplysia* glial cells as well[237]. We did not observe expression of orthologs of these genes in our dataset.

Cephalopod glia

Putative glia bearing morphological and physiological similarities to vertebrate glial cells have been described as early as 1896[238] and subsequently further described by Ramòn y Cajal (1917) [239], Barber and Graziadei[240] and JZ Young and collaborators[241–245].

These studies distinguish (1) glial cell associated blood vessels, (2) protoplasmic glia and (3) fibrous glia. In addition, Gray also described enigmatic dark cells that may be support cells[246].

Glial cells associated with blood vessels were reported to be attached to perivascular spaces of blood vessels. Stephens & Young suggested that glial cells perform a nourishment or support function making up a "glio-vascular system" which resembled "grape-like" clusters of "lymphoid" spaces around venous channels and act as a drainage to the venous system[242]. These spaces were described to also contain collagen, smooth muscle cells and fibroblasts[246]. Similar observations were made in the cuttlefish *Sepia officinalis*[247,248] where although the glio-vascular system was reported to be less prominent. There has however been some controversy over whether these glial cells contribute to the blood-brain barrier. Barber & Graziadei originally observed that neither endothelial, pericyte cells or perivascular glia fully completely insulated the vascular system and concluded that there was no true blood-brain barrier in cephalopods[240]. However a subsequent study by Bundgaard and Abbot (1992) in *Sepia* demonstrated a "seamless" perivascular glial layer of endfeet separating all neuronal cells except for neurosecretory cells[248]. The same observations were later made in other coleoid cephalopods (squid, octopus) [247]. Furthermore, axons connecting the *Sepia* optic lobe and retina were permeable to horseradish peroxidase[247].

Protoplasmic astrocyte-like glia were initially described by Cajal and Borogaze & Cazal[239,249]. These glial cells have endfeet attached to blood vessels and to glio-vascular cells. Stephens & Young observed that protoplasmic glia form sheaths around the whole nerve cell body and fibres[242].

Fibrous neuroglia contain more glial fibrils than protoplasmic glia more related to fibre tracts. Huge cells with many fibres arising from the centre. Along the neuropil away from blood vessels or glio-vascular strands. These cells have been described to be located in the plexiform zone of cells in bundles.

We observe expression of Eaa1-2 glial marker in both large cells situated in the palisade layer of the medulla (see Fig. 3j-j', arrowheads) and in a punctate pattern in the inner plexiform layer. We propose that the larger cells may correspond to fibrous glia, whereas the punctate pattern may correspond to protoplasmic glia. Case, Gray and Young showed that the basement membrane separating the plexiform and outer granular layer of the optic lobe in *Eledone* and Octopus was composed of a sheet of flattened glial processes[250].

The cephalopod retina is known to contain enigmatic support cells that some have hypothesised to be glial in nature[251]. We do not recover any of the glial markers observed in the optic lobes in the retinal support cells. Instead we believe that these cells are more closely related to photoreceptor cell types.

Supplementary References

1. Bacq, Z. M. Recherches sur la physiologie et la pharmacologie du système nerveux autonome. XVII. Les esters de la choline dans les extraits de tissus des invertébrés. *Arch. Int. Physiol.* **42**, 24 (1935).
2. Bacq, Z. M. & Mazza, F. P. *Identification D'acétylcholine Extraite Des Cellules Ganglionnaires d'Octopus*. (Masson, 1935).
3. Corteggiani, E. *Contribution à L'étude de L'acétylcholine Libre et Dissimulée Sous Forme D'un Complexe Dans Le Cerveau*. (Ch. Dessaint, 1938).
4. Heilbronn, E., Hause, S. & Lundgren, G. Chemical identification of acetylcholine in squid-head ganglion. *Brain Res.* **33**, 431–437 (1971).
5. Dowdall, M. J. & Whittaker, V. P. Comparative studies in synaptosome formation: the preparation of synaptosomes from the head ganglion of the squid, Loligo pealii. *J. Neurochem.* **20**, 921–935 (1973).
6. Loe, P. R. & Florey, E. The distribution of acetylcholine and cholinesterase in the nervous system and in innervated organs of Octopus dofleini. *Comp. Biochem. Physiol.* **17**, 509–522 (1966).
7. Jones, D. G. An electron-microscope study of subcellular fractions of Octopus brain. *J. Cell Sci.* **2**, 573–586 (1967).
8. Florey, E. & Winesdorfer, J. Cholinergic nerve endings in octopus brain. *J. Neurochem.* **15**, 169–177 (1968).
9. Martin, R., Barlow, J. & Miralto, A. Application of the zinc iodide-osmium tetroxide impregnation of synaptic vesicles in cephalopod nerves. *Brain Res.* **15**, 1–16 (1969).
10. Florey, E. & Florey, E. Über die mögliche Bedeutung von Enteramin (5-Oxy-Tryptamin) als nervöser Aktionssubstanz bei Cephalopoden und dekapoden Crustaceen. *Zeitschrift für Naturforschung B* **9**, 58–68 (1954).
11. Welsch, F. & Dettbarn, W.-D. The subcellular distribution of acetylcholine, cholinesterases and choline acetyltransferase in optic lobes of the squidLoligo pealei. *Brain Res.* **39**, 467–482 (1972).
12. D'Este, L. *et al.* First visualization of cholinergic cells and fibers by immunohistochemistry for choline acetyltransferase of the common type in the optic lobe and peduncle complex of Octopus vulgaris. *J. Comp. Neurol.* **509**, 566–579 (2008).
13. Casini, A. *et al.* Immunolocalization of choline acetyltransferase of common type in the central brain mass of Octopus vulgaris. *Eur. J. Histochem.* **56**, e34 (2012).
14. Pungor, J. R. Characterization of the Visual System of Octopus bimaculoides. (search.proquest.com, 2014).
15. Songco-Casey, J. O. *et al.* Cell types and molecular architecture of the Octopus bimaculoides visual system. *Curr. Biol.* (2022) doi:10.1016/j.cub.2022.10.015.
16. Nachmansohn, D. & Weiss, M. S. STUDIES ON CHOLINE ACETYLASE: IV. EFFECT OF CITRIC ACID. *J. Biol. Chem.* **172**, 677–687 (1948).
17. Lam, D. M., Wiesel, T. N. & Kaneko, A. Neurotransmitter synthesis in cephalopod retina. *Brain Res.* **82**, 365–368 (1974).
18. Bellanger, C., Dauphin, F., Belzunces, L. P., Cancian, C. & Chichery, R. Central acetylcholine synthesis and catabolism activities in the cuttlefish during aging. *Brain Res.* **762**, 219–222 (1997).
19. Silva, V. S. *et al.* Comparative effects of aluminum and ouabain on synaptosomal choline uptake, acetylcholine release and (Na+/K+)ATPase. *Toxicology* **236**, 158–177 (2007).
20. Nunes, M. A. *et al.* Acetylcholine release and choline uptake by cuttlefish (Sepia officinalis) optic lobe synaptosomes. *Biol. Bull.* **214**, 1–5 (2008).
21. Husain, S. S. & Mautner, H. G. The purification of choline acetyltransferase of squid-head ganglia. *Proc. Natl. Acad. Sci. U. S. A.* **70**, 3749–3753 (1973).
22. Prempeh, A. B., Prince, A. K. & Hide, E. G. The reaction of acetyl-coenzyme A with choline acetyltransferase. *Biochem. J* **129**, 991–994 (1972).
23. Korkes, S. *et al.* Coupling of acetyl donor systems with choline acetylase. *J. Biol. Chem.* **198**, 215–220 (1952).
24. Berman, R., Wilson, I. B. & Nachmansohn, D. Choline acetylase specificity in relation to biological function. *Biochim. Biophys. Acta* **12**, 315–324 (1953).
25. Korey, S. R., de BRAGANZA, B. & Nachmansohn, D. Choline acetylase. V. Esterifications and transacetylations. *J. Biol. Chem.* **189**, 705–715 (1951).
26. Prince, A. K. Properties of choline acetyltransferase isolated from squid ganglia. *Proc. Natl. Acad. Sci. U. S. A.* **57**, 1117–1122 (1967).
27. Feldberg, W., Harris, F. W. & Lin, R. C. Y. Observations on the presence of cholinergic and non-cholinergic neurones in the central nervous system. *J. Physiol.* **112**, 400–404 (1951).
28. Dowdall, M. J. & Simon, E. J. Comparative studies on synaptosomes: uptake of (N-Me-3H)choline by synaptosomes from squid optic lobes. *J. Neurochem.* **21**, 969–982 (1973).
29. Barker, L. A., Dowdall, M. J., Vickers, G. R. & Mittag, T. W. HIGH-AFFINITY CHOLINE TRANSPORT-UPTAKE AND METABOLISM OF CHOLINE AND PYRROLE CHOLINE BY SYNAPTOSOMES FROM OPTIC LOBE OF SQUID (LOLIGO-PEALEI). in *BIOLOGICAL BULLETIN* vol. 147 468–468 (MARINE BIOLOGICAL LABORATORY 7 MBL ST, WOODS HOLE, MA 02543, 1974).
30. Pollard, H. B., Barker, J. L., Bohr, W. A. & Dowdall, M. J. Chlorpromazine: Specific inhibition of L-noradrenaline and 5-hydroxytryptamine uptake in synaptosomes from squid brain. *Brain Res.* **85**, 23–31 (1975).
31. Hoskin, F. C. & Long, R. J. Purification of a DFP-hydrolyzing enzyme from squid head ganglion. *Arch. Biochem. Biophys.* **150**, 548–555 (1972).
32. Drukker, J. & Schadé, J. P. Neurobiological studies on cephalopods III. Histochemistry of 24 enzymes in the optic system. *Neth. J. Sea Res.* **2**, 155–182 (1964).
33. Barlow, J. J. Comparative biochemistry of the central nervous system. *Symp Zool Soc Lond* (1977).

34. Nachmansohn, D. & Meyerhof, B. Relation between electrical changes during nerve activity and concentration of choline esterase. *J. Neurophysiol.* **4**, 348–361 (1941).
35. Chichery, M. P. & Chichery, R. Histochemical study of the localization of cholinesterases in the central nervous system of Sepia officinalis. *Cell Tissue Res.* **148**, 551–560 (1974).
36. Bellanger, C., Dauphin, F., Chichery, M.-P. & Chichery, R. Changes in cholinergic enzyme activities in the cuttlefish brain during memory formation. *Physiol. Behav.* **79**, 749–756 (2003).
37. Кулиева, А. М. , Розенгарт , В. И. и . Шмелева, В. Г [Kulieva A. M., Rozengart, V. I. & Shmeleva V. G.]. Некоторые особенности структуры активной поверхности холинэстеразы зрительного ганглия кальмара, [English title: Structural characteristics of the active surface of cholinesterase in the squid optic ganglion]. *Биохимия [Biokhimiya]* **36,** 568–571 (1971).
38. Боголюбова, Г.М. , Карпинская, Е.В. , Куликова, А.И. , Розенгарт, В.И. [Bogolubova, G.M, Karpinskaya, E.V., Kulikova, A.I., Rozengart, V.I,]. Субстратная специфичность холинэстеразы зрительных ганглиев тихоокеанского кальмара и ацетилхолинэстеразы эритроцитов быка [English title: Substrate specificity of choline esterase from optic ganglia of calamary and acetyl choline esterase from beef erythrocyte]. *Биохимия [Biokhimiya]* **37**, 826–833 (1972).
39. Куприянов, В. А., Куликова, А. И. и Розенгарт , В. И. [Kupriyankov, V.A, Kulikova, A.I, Rozengart, V.I,]. Кинетика ингибирования аммониевыми соединениями гидролиза различных субстратов холинэстеразой из зрительных ганглиев кальмара, [English Title: Kinetics of inhibition by ammonnium compounds of hydrolysis of various substrates under the action of choline esterase from the optical ganglion of the squid]. *Биохимия [Biokhimiya]* **38**, 1261–1266 (1973).
40. Tansey, E. M. Neurotransmitters in the cephalopod brain. *Comp. Biochem. Physiol. C Pharmacol. Toxicol. Endocrinol.* **64**, 173–182 (1979).
41. Kato, G. & Tattrie, B. Studies on the Cholinergic Receptor of Squid Optic Ganglia. in *Molecular and Quantum Pharmacology* 189–209 (Springer Netherlands, 1974). doi:10.1007/978-94-010-1758-9_14.
42. Turpaev, T. M. *et al.* Cholinesterase of squid optical ganglia. *Eur. J. Biochem.* **6**, 55–59 (1968).
43. Tasaki & Tsukahara. 2 TYPES OF INHIBITION IN THE CEPHALOPOD RETINA. *Biomed. saf. stand.* (1982).
44. Albertin, C. B. *et al.* The octopus genome and the evolution of cephalopod neural and morphological novelties. *Nature* **524**, 220–224 (2015).
45. Andrews, P. L., Messenger, J. B. & Tansey, E. M. Colour changes in cephalopods after neurotransmitter injection into the cephalic aorta. *Proc. R. Soc. Lond. B Biol. Sci.* **213**, 93–99 (1981).
46. Piscopo, S. *et al.* Pre- and postsynaptic excitation and inhibition at octopus optic lobe photoreceptor terminals; implications for the function of the ‘presynaptic bags’. *Eur. J. Neurosci.* **26**, 2196–2203 (2007).
47. Демушкин, В.П. , Котелевцев, Ю.В. [Demushkin, V.P., Kotelevtsev, Y.V.]. Свойства мембраносвязанного ацетилхолинового рецептора из зрительных ганглиев кальмара Berryteuthis magister [English title:1980. Properties of membrane-bound acetylcholine receptor from optic ganglia of the squid Berryteuthis magister.]. *Биохимия [Biokhimiya]* **45**, 1773–1779 (1980).
48. Демушкин, В.П., Котелевцев, Ю. В. [Demushkin, V.P, Kotelevtsev, Y.V.,]. Фотоаффинная модификация азидоцитизином никотинового ацетилхолинового рецептора из зрительных ганглиев кальмара [English title: Azidocytisine photoaffinity labeling of the nicotinic acetylcholine receptor from the squid optic ganglia]. *Bioorg. Khim* **8**, 621–629 (1982).
49. Gennad’evich], П. Ю. Г. [plyashkevich. Структурные особенности участка узнавания холинергических лигандов нАХР зрительных ганглиев кальмара [English title: Structural specificities in the field of cholinergic ligands of nicotinic acetylcholine receptors of optical ganglia of squid]. (Научно-исследовательский институт мозга всесоюзного научного центра психического здоровья академии медицинских наук СССР, 1985).
50. Chen, S. J., Spathis, R. & Schmidt, J. Binding sites for [3H]-acetylcholine and 125I-alpha-bungarotoxin in the optic ganglion of Loligo pealii. *Comp. Biochem. Physiol. C* **90**, 317–323 (1988).
51. Chrachri, A. & Williamson, R. Cholinergic and glutamatergic spontaneous and evoked excitatory postsynaptic currents in optic lobe neurons of cuttlefish, Sepia officinalis. *Brain Res.* **1020**, 178–187 (2004).
52. Bellanger, C., Halm, M.-P., Dauphin, F. & Chichery, R. In vitro evidence and age-related changes for nicotinic but not muscarinic acetylcholine receptors in the central nervous system of Sepia officinalis. *Neurosci. Lett.* **387**, 162–167 (2005).
53. Cory, H. T. & Rose, S. P. R. Glucose and amino acid metabolism in octopus optic and vertical lobes in vitro. *J. Neurochem.* **16**, 979–988 (1969).
54. D’Aniello, A. *et al.* Free l-amino acids and d-aspartate content in the nervous system of Cephalopoda. A comparative study. *Comp. Biochem. Physiol. B Biochem. Mol. Biol.* **112**, 661–666 (1995).
55. D’aniello, S. *et al.* Cephalopod vision involves dicarboxylic amino acids: D-aspartate, L-aspartate and L-glutamate. *Biochem. J* **386**, 331–340 (2005).
56. Tsukada, Y., Takagaki, G., Sugimoto, S. & Hirano, S. Changes in the ammonia and glutamine content of the rat brain induced by electric shock. *J. Neurochem.* **2**, 295–303 (1958).
57. Tsukada, Y., Uemura, K., Hirano, S. & Nagata, Y. Distribution of amino acids in the brain in different species. in *Comparative neurochemistry* 179–183 (Elsevier, 1964).
58. Kleinschuster, S. J. & Morris, J. E. Glutamine synthetase, an enzyme characteristic of vertebrate systems in invertebrate tissues. *Experientia* **28**, 1157–1158 (1972).
59. Di Cosmo, A., Paolucci, M. & Di Cristo, C. N-methyl-D-aspartate receptor-like immunoreactivity in the brain of Sepia and Octopus. *J. Comp. Neurol.* **477**, 202–219 (2004).
60. Battaglia, A. A. *et al.* Cloning and characterization of an ionotropic glutamate receptor subunit expressed in the squid nervous system. *Eur. J. Neurosci.* **17**, 2256–2266 (2003).

61. Lima, P. A., Nardi, G. & Brown, E. R. AMPA/kainate and NMDA-like glutamate receptors at the chromatophore neuromuscular junction of the squid: role in synaptic transmission and skin patterning. *Eur. J. Neurosci.* **17**, 507–516 (2003).
62. Kwon, K.-M., Pak, J.-H. & Jeon, C.-J. Immunocytochemical localization of the AMPA glutamate receptor subtype GluR2/3 in the squid optic lobe. *Acta Histochem.* **124**, 151941 (2022).
63. Palumbo, A., Di Cosmo, A., Poli, A., Di Cristo, C. & d'Ischia, M. A calcium/calmodulin-dependent nitric oxide synthase, NMDAR2/3 receptor subunits, and glutamate in the CNS of the cuttlefish Sepia officinalis: localization in specific neural pathways controlling the inking system. *J. Neurochem.* **73**, 1254–1263 (1999).
64. Palumbo, A., Fiore, G., Di Cristo, C., Di Cosmo, A. & d'Ischia, M. NMDA receptor stimulation induces temporary α-tubulin degradation signaled by nitric oxide-mediated tyrosine nitration in the nervous system of Sepia officinalis. *Biochem. Biophys. Res. Commun.* **293**, 1536–1543 (2002).
65. Chichery, R. & Chichery, M. P. NADPH-diaphorase in a cephalopod brain (Sepia): presence in an analogue of the cerebellum. *Neuroreport* **5**, 1273–1276 (1994).
66. Moroz, L. L., Chen, D., Gillette, M. U. & Gillette, R. Nitric oxide synthase activity in the molluscan CNS. *J. Neurochem.* **66**, 873–876 (1996).
67. Kimura, T., Shouno, O. & Matsumoto, G. NADPH-diaphorase containing cells and fibers in the central nervous system of squid, Loligo bleekeri keferstein. *Life Sci.* **61**, 2375–2381 (1997).
68. Scheinker, V. *et al.* Nitric oxide synthase in the nervous system and ink gland of the cuttlefish Sepia officinalis: molecular cloning and expression. *Biochem. Biophys. Res. Commun.* **338**, 1204–1215 (2005).
69. Di Cosmo, A., Di Cristo, C., Palumbo, A., d'Ischia, M. & Messenger, J. B. Nitric oxide synthase (NOS) in the brain of the cephalopod Sepia officinalis. *J. Comp. Neurol.* **428**, 411–427 (2000).
70. Di Cristo, C. *et al.* Nitric oxide synthase expression in the central nervous system of Sepia officinalis: an in situ hybridization study. *Eur. J. Neurosci.* **26**, 1599–1610 (2007).
71. Matus, A. I. Histochemical localization of biogenic monoamines in the cephalic ganglia of Octopus vulgaris. *Tissue Cell* **5**, 591–601 (1973).
72. Tansey, E. M. & Young, J. Z. Aminergic fluorescence in the cephalopod brain. *Philos. Trans. R. Soc. Lond. B Biol. Sci.* **291**, 127–145 (1980).
73. Silver, S. C., Patterson, J. A. & Mobbs, P. G. Biogenic amines in cephalopod retina. *Brain Res.* **273**, 366–368 (1983).
74. Juorio, A. V. & Molinoff, P. B. The normal occurrence of octopamine in neural tissue of the Octopus and other cephalopods. *J. Neurochem.* **22**, 271–280 (1974).
75. Makman, M. H., Berrios, I., Pratt, S., Hanhm, R. T. & Stefano, G. B. Anatomical localization of dopaminergic systems in Octopus retina; evidence for intrinsic dopamine-containing cells and dopamine D1 receptors. *Neurobiology: molluscan models. Amsterdam: North Holland* 31–35 (1987).
76. Suzuki, H. & Tasaki, K. Inhibitory retinal efferents from dopaminergic cells in the optic lobe of the octopus. *Vision Res.* **23**, 451–457 (1983).
77. Gleadall, I. G., Ohtsu, K., Gleadall, E. & Tsukahara, Y. Screening-pigment migration in the octopus retina includes control by dopaminergic efferents. *J. Exp. Biol.* **185**, 1–16 (1993).
78. Han, J.-M. & Chang, N.-S. Immuno-Electron Microscopic Studies on the Distribution of Dopamine and $ Calbindin-D_ ${28K} in the Optic lobes of Cephalopods (Todarodes pacificus and Octopus minor) inhabiting the Korean waters. *Applied Microscopy* **32**, 175–183 (2002).
79. Capasso, A. *et al.* A dopamine- and octopamine-sensitive adenylate cyclase in the nervous system of Octopus vulgaris. *Comp. Biochem. Physiol. B* **100**, 805–808 (1991).
80. Juorio, A. V. Catecholamines and 5-hydroxytryptamine in nervous tissue of cephalopods. *J. Physiol.* **216**, 213–226 (1971).
81. Kime, D. E. & Messenger, J. B. Monoamines in the cephalopod CNS: An HPLC analysis. *Comp. Biochem. Physiol. C Pharmacol. Toxicol. Endocrinol.* **96**, 49–57 (1990).
82. Martin, R. & Barlow, J. J. Changes in glial cells of the octopus brain after 6-hydroxydopamine administration. *Proc. R. Soc. Lond. B Biol. Sci.* **196**, 431–441 (1977).
83. Chang, N.-S. *et al.* Immuno-Electron Microscopic Studies on the Localization of Serotonin and Somatostatin in the Optic Lobes of Cephalopods (Todarodes pacificus and Octopus minor) Inhabiting the Korean Waters. *Applied Microscopy* **32**, 247–255 (2002).
84. Roberts, E. Comparative aspects of the distribution of ninhydrin-reactive constituents in nervous tissue. *Comparative Neurochemistry* (1964).
85. Cory, H. T. Comparative metabolic studies in octopus and rat brain. (1969).
86. Bradford, H. F., Chain, E. B., Cory, H. T. & Rose, S. P. Glucose and amino acid metabolism in some invertebrate nervous systems. *J. Neurochem.* **16**, 969–978 (1969).
87. Rose, S. P. & Cory, H. T. Glutamate metabolism in octopus brain in vivo; absence of a Waelsch effect. *J. Neurochem.* **17**, 817–820 (1970).
88. Osborne, N. N. Occurence of GABA and taurine in the nervous systems of the dogfish and some invertebrates. *Comp. Gen. Pharmacol.* **2**, 433–438 (1971).
89. Baret, R., Morgue, M., Broc, A. & Charmot, J. Etude comparative de la désamidination de l'acide γ-guanido-butyrique, et de l'arginine par l'hépatopancréas ou le foie de divers Invertébrés. *C. R. Seances Soc. Biol. Fil.* (1965).
90. Boldyrev, A. A. & Lebedev, A. V. Precursors of histidine dipeptides in molluscan tissues. *Comparative Biochemistry and Physiology Part B: Comparative Biochemistry* **41**, 453–456 (1972).
91. Cornwell, C. J., Messenger, J. B. & Williamson, R. Distribution of GABA-like immunoreactivity in the octopus brain. *Brain*

Res. **621**, 353–357 (1993).
92. Kobayashi, S., Takayama, C. & Ikeda, Y. Distribution of glutamic acid decarboxylase immunoreactivity within the brain of oval squid Sepioteuthis lessoniana. *Aquat. Biol.* **19**, 97–109 (2013).
93. Mann, E. & Enna, S. J. Phylogenetic distribution of bicuculline-sensitiveγ-amino-butyric acid (GABA) receptor binding. *Brain Res.* **184**, 367–373 (1980).
94. Chichery, R. & Chichery, M. P. Motor and behavioural effects induced by putative neurotransmitter injection into the optic lobe of the cuttlefish, Sepia officinalis. *Comp. Biochem. Physiol. C* **80**, 415–419 (1985).
95. Osborne, N. N. Occurrence of glycine and glutamic acid in the nervous system of two fish species and some invertebrates. *Comp. Biochem. Physiol. B* **43**, 579–585 (1972).
96. Gould, R. M. & Cottrell, G. A. Putrescine in molluscs: identification and occurrence in neurones and other tissues. *Comp. Biochem. Physiol. B* **48**, 591–597 (1974).
97. Bertaccini, G. A Discussion to H. H. Adam 'Histamine in the central nervous system' . in *Regional Neurochemistry* (ed. Elkes, S. S. K. A.) 305–306 (Pergamon Press, Oxford, 1961).
98. Scaros, A. T., Andouche, A., Baratte, S. & Croll, R. P. Histamine and histidine decarboxylase in the olfactory system and brain of the common cuttlefish Sepia officinalis (Linnaeus, 1758). *J. Comp. Neurol.* **528**, 1095–1112 (2020).
99. Elphick, M. R., Mirabeau, O. & Larhammar, D. Evolution of neuropeptide signalling systems. *J. Exp. Biol.* **221**, (2018).
100. Zatylny-Gaudin, C. *et al.* Neuropeptidome of the cephalopod Sepia officinalis: Identification, tissue mapping, and expression pattern of neuropeptides and neurohormones during egg laying. *J. Proteome Res.* **15**, 48–67 (2016).
101. Kamatani, Y. *et al.* Achatin-I, an endogenous neuroexcitatory tetrapeptide from Achatina fulica Férussac containing a D-amino acid residue. *Biochem. Biophys. Res. Commun.* **160**, 1015–1020 (1989).
102. Satake, H. *et al.* Characterization of a cDNA encoding a precursor polypeptide of a D-amino acid-containing peptide, achatin-I and localized expression of the achatin-I and fulicin genes. *Eur. J. Biochem.* **261**, 130–136 (1999).
103. Iwakoshi, E., Hisada, M. & Minakata, H. Cardioactive peptides isolated from the brain of a Japanese octopus, Octopus minor. *Peptides* **21**, 623–630 (2000).
104. Perez, D. M. L. Análisis funcional del control neuroendocrino del lóbulo óptico de las Hembras de Octopus maya bajo estrés térmico. (2021).
105. Thiel, D., Yañez-Guerra, L. A., Franz-Wachtel, M., Hejnol, A. & Jékely, G. Nemertean, Brachiopod, and Phoronid Neuropeptidomics Reveals Ancestral Spiralian Signaling Systems. *Mol. Biol. Evol.* **38**, 4847–4866 (2021).
106. Kuroki, Y. *et al.* A molluscan neuropeptide related to the crustacean hormone, RPCH. *Biochem. Biophys. Res. Commun.* **167**, 273–279 (1990).
107. ACM De Boer, P. & Maat, A. T. Functional Role of Peptidergic Anterior Lobe Neurons in Male Sexual Behavior of the Snail Lymnaea stagnalis. *Journal of* (1997).
108. Veenstra, J. A. Neuropeptide evolution: neurohormones and neuropeptides predicted from the genomes of Capitella teleta and Helobdella robusta. *Gen. Comp. Endocrinol.* **171**, 160–175 (2011).
109. Di Cristo, C., Van Minnen, J. & Di Cosmo, A. The presence of APGWamide in Octopus vulgaris: a possible role in the reproductive behavior. *Peptides* **26**, 53–62 (2005).
110. Henry, J., Favrel, P. & Boucaud-Camou, E. Isolation and identification of a novel Ala-Pro-Gly-Trp-amide-related peptide inhibiting the motility of the mature oviduct in the cuttlefish, Sepia officinalis. *Peptides* **18**, 1469–1474 (1997).
111. Henry, J. & Zatylny, C. Identification and tissue mapping of APGWamide-related peptides in Sepia officinalis using LC-ESI-MS/MS. *Peptides* **23**, 1031–1037 (2002).
112. Sirinupong, P., Suwanjarat, J. & van Minnen, J. Distribution of APGWamide-immunoreactivity in the brain and reproductive organs of adult pygmy squid, Idiosepius pygmaeus. *Invert. Neurosci.* **11**, 97–102 (2011).
113. Veenstra, J. A. Neurohormones and neuropeptides encoded by the genome of Lottia gigantea, with reference to other mollusks and insects. *Gen. Comp. Endocrinol.* **167**, 86–103 (2010).
114. Dewey, E. M. *et al.* Identification of the gene encoding bursicon, an insect neuropeptide responsible for cuticle sclerotization and wing spreading. *Curr. Biol.* **14**, 1208–1213 (2004).
115. Suzuki, H. & Yamamoto, T. Centrifugal neurons of the octopus optic lobe cortex are immunopositive for calcitonin gene-related peptide. *Neurosci. Lett.* **324**, 21–24 (2002).
116. Altobelli, G. G., Van Noorden, S. & Cimini, V. Calcium-binding protein and some neuropeptides in the retina of Octopus vulgaris : A morpho-histochemical study. *J. Cell. Physiol.* **233**, 6866–6876 (2018).
117. Lafont, A.-G., Dufour, S. & Fouchereau-Peron, M. Evidence for the presence of molecules related to the neuropeptide CGRP in two cephalopods, Sepia officinalis and Nautilus macromphalus: comparison with its target organ distribution. *Neuroendocrinology* **84**, 138–150 (2006).
118. Ikeda, T., Minakata, H., Fujita, T., Muneoka, Y. & Kiss, T. Neuropeptides isolated from Helix pomatia Part 1. Peptides related to MIP, buccalin, myomodulin-CARP and SCP. *Chemistry* (1992).
119. Ponesakki, V. *et al.* Annotation of nerve cord transcriptome in earthworm Eisenia fetida. *Genom Data* **14**, 91–105 (2017).
120. De Oliveira, A. L., Calcino, A. & Wanninger, A. Extensive conservation of the proneuropeptide and peptide prohormone complement in mollusks. *Sci. Rep.* **9**, 4846 (2019).
121. Thiel, D., Bauknecht, P., Jékely, G. & Hejnol, A. A nemertean excitatory peptide/CCHamide regulates ciliary swimming in the larvae of Lineus longissimus. *Front. Zool.* **16**, 28 (2019).
122. Zieger, E., Robert, N. S. M., Calcino, A. & Wanninger, A. Ancestral Role of Ecdysis-Related Neuropeptides in Animal Life Cycle Transitions. *Curr. Biol.* **31**, 207–213.e4 (2021).
123. Lenaerts, C. *et al.* The ecdysis triggering hormone system is essential for successful moulting of a major hemimetabolous

pest insect, Schistocerca gregaria. *Sci. Rep.* **7**, 46502 (2017).

124. Suzuki, H., Muraoka, T. & Yamamoto, T. Localization of corticotropin-releasing factor-immunoreactive nervous tissue and colocalization with neuropeptide Y-like substance in the optic lobe and peduncle complex of the octopus (Octopus vulgaris). *Cell Tissue Res.* **313**, 129–138 (2003).
125. Amano, M., Oka, Y., Nagai, Y., Amiya, N. & Yamamori, K. Immunohistochemical localization of a GnRH-like peptide in the brain of the cephalopod spear-squid, Loligo bleekeri. *Gen. Comp. Endocrinol.* **156**, 277–284 (2008).
126. El Filali, Z., Van Minnen, J., Liu, W. K., Smit, A. B. & Li, K. W. Peptidomics analysis of neuropeptides involved in copulatory behavior of the mollusk Lymnaea stagnalis. *J. Proteome Res.* **5**, 1611–1617 (2006).
127. Mitsukawa, K., Lu, X. & Bartfai, T. Galanin, galanin receptors and drug targets. *Cell. Mol. Life Sci.* **65**, 1796–1805 (2008).
128. Suzuki, H., Yamamoto, T., Inenaga, M. & Uemura, H. Galanin-immunoreactive neuronal system and colocalization with serotonin in the optic lobe and peduncle complex of the octopus (Octopus vulgaris). *Brain Res.* **865**, 168–176 (2000).
129. Zatylny-Gaudin, C. *et al.* Characterization of a novel LFRFamide neuropeptide in the cephalopod Sepia officinalis. *Peptides* **31**, 207–214 (2010).
130. Zhang, Z. & Tublitz, N. J. Expression of the SOFaRP2 gene in the central nervous system of the adult cuttlefish Sepia officinalis. *Neuropeptides* **47**, 149–155 (2013).
131. Cao, Z.-H. *et al.* Molecular cloning, expression analysis and cellular localization of an LFRFamide gene in the cuttlefish Sepiella japonica. *Peptides* **80**, 40–47 (2016).
132. Suzuki, H., Yamamoto, T., Nakagawa, M. & Uemura, H. Neuropeptide Y-immunoreactive neuronal system and colocalization with FMRFamide in the optic lobe and peduncle complex of the octopus (Octopus vulgaris). *Cell Tissue Res.* **307**, 255–264 (2002).
133. Smart, D. *et al.* Peptide tyrosine phenylalanine: a novel neuropeptide F-related nonapeptide from the brain of the squid, Loligo vulgaris. *Biochem. Biophys. Res. Commun.* **186**, 1616–1623 (1992).
134. Stangier, J., Hilbich, C., Burdzik, S. & Keller, R. Orcokinin: a novel myotropic peptide from the nervous system of the crayfish, Orconectes limosus. *Peptides* **13**, 859–864 (1992).
135. Li, G. *et al.* Molecular characterization, expression and localization analysis of a pedal like peptide in cuttlefish Sepiella japonica. *Research Square* (2022) doi:10.21203/rs.3.rs-1640075/v1.
136. Qiu, J.-Y., Zheng, L.-B. & Chi, C.-F. Identification, Characterization, and Expression of a PRQFVamide-Related Peptide in Cephalopod Sepiella japonica. *Frontiers in Marine Science* **9**, (2022).
137. Feldman, S. C. Distribution of immunoreactive somatostatin (ISRIF) in the nervous system of the squid, Loligo pealei. *J. Comp. Neurol.* **245**, 238–257 (1986).
138. Feldman, S. C. Immunohistochemical localization of somatostatin and calcium-binding protein in squid and Aplysia neurons. *Federation Proceedings of the Federation of American Societies for Experimental Biology* **41**, 1955 (1982).
139. Bardou, I. *et al.* Distribution of oxytocin-like and vasopressin-like immunoreactivities within the central nervous system of the cuttlefish, Sepia officinalis. *Cell Tissue Res.* **336**, 249–266 (2009).
140. Henry, J., Cornet, V., Bernay, B. & Zatylny-Gaudin, C. Identification and expression of two oxytocin/vasopressin-related peptides in the cuttlefish Sepia officinalis. *Peptides* **46**, 159–166 (2013).
141. Bardou, I. *et al.* Ontogeny of oxytocin-like immunoreactivity in the cuttlefish, Sepia officinalis, central nervous system. *Dev. Neurosci.* **32**, 19–32 (2010).
142. Duruz, J. *et al.* Molecular characterization of cell types in the squid Loligo vulgaris. *Elife* **12**, (2023).
143. Styfhals, R. *et al.* Cell type diversity in a developing octopus brain. *Nat. Commun.* **13**, 7392 (2022).
144. Somjen, G. G. Nervenkitt: notes on the history of the concept of neuroglia. *Glia* **1**, 2–9 (1988).
145. Pentreath, V. W. Invertebrate glial cells. *Comp. Biochem. Physiol. A Physiol.* **93**, 77–83 (1989).
146. Coles, J. A. Functions of glial cells in the retina of the honeybee drone. *Glia* **2**, 1–9 (1989).
147. Shaham, S. Glia-neuron interactions in nervous system function and development. *Curr. Top. Dev. Biol.* **69**, 39–66 (2005).
148. Yildirim, K., Petri, J., Kottmeier, R. & Klämbt, C. Drosophila glia: Few cell types and many conserved functions: YILDIRIM et al. *Glia* **67**, 5–26 (2019).
149. Raff, M. C. Glial cell diversification in the rat optic nerve. *Science* **243**, 1450–1455 (1989).
150. Verkhratsky, A., Ho, M. S. & Parpura, V. Evolution of Neuroglia. *Adv. Exp. Med. Biol.* **1175**, 15–44 (2019).
151. Hartline, D. K. The evolutionary origins of glia. *Glia* **59**, 1215–1236 (2011).
152. Bronner, M. E. & LeDouarin, N. M. Development and evolution of the neural crest: an overview. *Dev. Biol.* **366**, 2–9 (2012).
153. Markey, K. M., Saunders, J. C., Smuts, J., von Reyn, C. R. & Garcia, A. D. R. Astrocyte development—More questions than answers. *Frontiers in Cell and Developmental Biology* **11**, (2023).
154. Liu, B., Hunter, D. J., Smith, A. A., Chen, S. & Helms, J. A. The capacity of neural crest-derived stem cells for ocular repair: Neural Crest-Derived Stem Cells for Ocular Repair. *Birth Defects Res. C Embryo Today* **102**, 299–308 (2014).
155. Sharma, G. & Vijayaraghavan, S. Nicotinic receptor signaling in nonexcitable cells. *J. Neurobiol.* **53**, 524–534 (2002).
156. Pan, S., Mayoral, S. R., Choi, H. S., Chan, J. R. & Kheirbek, M. A. Preservation of a remote fear memory requires new myelin formation. *Nat. Neurosci.* **23**, 487–499 (2020).
157. Liu, J. *et al.* Clemastine enhances myelination in the prefrontal cortex and rescues behavioral changes in socially isolated mice. *J. Neurosci.* **36**, 957–962 (2016).
158. Steadman, P. E. *et al.* Disruption of oligodendrogenesis impairs memory consolidation in adult mice. *Neuron* **105**, 150–164.e6 (2020).
159. Foerster, S. *et al.* Developmental origin of oligodendrocytes determines their function in the adult brain. *Nat. Neurosci.* **27**,

1545–1554 (2024).
160. Huang, W. *et al.* Origins and proliferative states of human oligodendrocyte precursor cells. *Cell* **182**, 594–608.e11 (2020).
161. Solovieva, T. & Bronner, M. Schwann cell precursors: Where they come from and where they go. *Cells Dev.* **166**, 203686 (2021).
162. MacDonald, A. *et al.* Single cell transcriptomics of ependymal cells across age, region and species reveals cilia-related and metal ion regulatory roles as major conserved ependymal cell functions. *Front. Cell. Neurosci.* **15**, 703951 (2021).
163. Nagy, N. & Goldstein, A. M. Enteric nervous system development: A crest cell's journey from neural tube to colon. *Semin. Cell Dev. Biol.* **66**, 94–106 (2017).
164. Rao, M. *et al.* Enteric glia express proteolipid protein 1 and are a transcriptionally unique population of glia in the mammalian nervous system. *Glia* **63**, 2040–2057 (2015).
165. Kierdorf, K. & Prinz, M. Microglia in steady state. *J. Clin. Invest.* **127**, 3201–3209 (2017).
166. Reemst, K., Noctor, S. C., Lucassen, P. J. & Hol, E. M. The Indispensable Roles of Microglia and Astrocytes during Brain Development. *Front. Hum. Neurosci.* **10**, 566 (2016).
167. Gomez Perdiguero, E. *et al.* Tissue-resident macrophages originate from yolk-sac-derived erythro-myeloid progenitors. *Nature* **518**, 547–551 (2015).
168. Kierdorf, K. *et al.* Microglia emerge from erythromyeloid precursors via Pu.1- and Irf8-dependent pathways. *Nat. Neurosci.* **16**, 273–280 (2013).
169. Borst, K., Dumas, A. A. & Prinz, M. Microglia: Immune and non-immune functions. *Immunity* **54**, 2194–2208 (2021).
170. Rey, S., Zalc, B. & Klämbt, C. Evolution of glial wrapping: A new hypothesis. *Dev. Neurobiol.* **81**, 453–463 (2021).
171. Garm, A., Poussart, Y., Parkefelt, L., Ekström, P. & Nilsson, D.-E. The ring nerve of the box jellyfish Tripedalia cystophora. *Cell Tissue Res.* **329**, 147–157 (2007).
172. Busengdal, H. & Rentzsch, F. Unipotent progenitors contribute to the generation of sensory cell types in the nervous system of the cnidarian Nematostella vectensis. *Dev. Biol.* **431**, 59–68 (2017).
173. Sheloukhova, L. & Watanabe, H. Analysis of cnidarian Gcm suggests a neuronal origin of glial EAAT1 function. *Sci. Rep.* **13**, (2023).
174. Sheloukhova, L. & Watanabe, H. Evolution of glial cells: a non-bilaterian perspective. *Neural Dev.* **19**, 10 (2024).
175. Sebé-Pedrós, A. *et al.* Cnidarian Cell Type Diversity and Regulation Revealed by Whole-Organism Single-Cell RNA-Seq. *Cell* **173**, 1520–1534.e20 (2018).
176. Bery, A., Cardona, A., Martinez, P. & Hartenstein, V. Structure of the central nervous system of a juvenile acoel, Symsagittifera roscoffensis. *Dev. Genes Evol.* **220**, 61–76 (2010).
177. Bedini, C., Ferrero, E. & Lanfranchi, A. The ultrastructure of ciliary sensory cells in two Turbellaria Acoela. *Tissue Cell* **5**, 359–372 (1973).
178. Duruz, J. *et al.* Acoel single-cell transcriptomics: Cell type analysis of a deep branching bilaterian. *Mol. Biol. Evol.* **38**, 1888–1904 (2021).
179. Søviknes, A. M. & Glover, J. C. Spatiotemporal patterns of neurogenesis in the appendicularian Oikopleura dioica. *Dev. Biol.* **311**, 264–275 (2007).
180. Meinertzhagen, I. A., Lemaire, P. & Okamura, Y. The neurobiology of the ascidian tadpole larva: recent developments in an ancient chordate. *Annu. Rev. Neurosci.* **27**, 453–485 (2004).
181. Bullock, T., Horridge, G., Bern, H., Hagedorn, I. R. & Smith, J. E. Structure and function in the nervous systems of invertebrates. *pp* (1965).
182. Lane, N. J. Neurosecretory cells in the cerebral ganglion of adult tunicates: fine structure and distribution of phosphatases. *J. Ultrastruct. Res.* **40**, 480–497 (1972).
183. Ryan, K., Lu, Z. & Meinertzhagen, I. A. The CNS connectome of a tadpole larva of Ciona intestinalis (L.) highlights sidedness in the brain of a chordate sibling. *Elife* **5**, e16962 (2016).
184. Sharma, S., Wang, W. & Stolfi, A. Single-cell transcriptome profiling of the Ciona larval brain. *Dev. Biol.* **448**, 226–236 (2019).
185. Medina, B. N. S. P. *et al.* Identification of astrocyte-like cells in an adult ascidian during regeneration of the central nervous system. *Glia* **72**, 2190–2200 (2024).
186. Mashanov, V. & Zueva, O. Radial Glia in Echinoderms. *Dev. Neurobiol.* **79**, 396–405 (2019).
187. San Miguel-Ruiz, J. E., Maldonado-Soto, A. R. & García-Arrarás, J. E. Regeneration of the radial nerve cord in the sea cucumber Holothuria glaberrima. *BMC Dev. Biol.* **9**, 3 (2009).
188. Mashanov, V. S., Zueva, O. R. & García-Arrarás, J. E. Radial glial cells play a key role in echinoderm neural regeneration. *BMC Biol.* **11**, 49 (2013).
189. Oikonomou, G. & Shaham, S. The glia of Caenorhabditis elegans. *Glia* **59**, 1253–1263 (2011).
190. Mizeracka, K. & Heiman, M. G. The many glia of a tiny nematode: studying glial diversity using Caenorhabditis elegans. *Wiley Interdiscip. Rev. Dev. Biol.* **4**, 151–160 (2015).
191. Stout, R. F., Jr, Verkhratsky, A. & Parpura, V. Caenorhabditis elegans glia modulate neuronal activity and behavior. *Front. Cell. Neurosci.* **8**, 67 (2014).
192. Fung, W., Wexler, L. & Heiman, M. G. Cell-type-specific promoters for C. elegans glia. *J. Neurogenet.* **34**, 335–346 (2020).
193. Hartenstein, V. Morphological diversity and development of glia in Drosophila. *Glia* **59**, 1237–1252 (2011).
194. Croset, V., Treiber, C. D. & Waddell, S. Cellular diversity in the Drosophila midbrain revealed by single-cell transcriptomics. *Elife* **7**, (2018).

195. Konstantinides, N. *et al.* Phenotypic Convergence: Distinct Transcription Factors Regulate Common Terminal Features. *Cell* **174**, 622–635.e13 (2018).
196. Lago-Baldaia, I. *et al.* A Drosophila glial cell atlas reveals a mismatch between transcriptional and morphological diversity. *PLoS Biol.* **21**, e3002328 (2023).
197. Kurmangaliyev, Y. Z., Yoo, J., Valdes-Aleman, J., Sanfilippo, P. & Zipursky, S. L. Transcriptional Programs of Circuit Assembly in the Drosophila Visual System. *Neuron* **108**, 1045–1057.e6 (2020).
198. Özel, M. N. *et al.* Neuronal diversity and convergence in a visual system developmental atlas. *Nature* **589**, 88–95 (2021).
199. Hosoya, T., Takizawa, K., Nitta, K. & Hotta, Y. glial cells missing: a binary switch between neuronal and glial determination in Drosophila. *Cell* **82**, 1025–1036 (1995).
200. Davie, K. *et al.* A single-cell transcriptome atlas of the aging Drosophila brain. *Cell* **174**, 982–998.e20 (2018).
201. Brunet Avalos, C., Maier, G. L., Bruggmann, R. & Sprecher, S. G. Single cell transcriptome atlas of the Drosophila larval brain. *Elife* **8**, e50354 (2019).
202. Ariss, M. M., Islam, A. B. M. M. K., Critcher, M., Zappia, M. P. & Frolov, M. V. Single cell RNA-sequencing identifies a metabolic aspect of apoptosis in Rbf mutant. *Nat. Commun.* **9**, 5024 (2018).
203. Fahrenbach, W. H. The brain of the horseshoe crab (Limulus polyphemus). I. Neuroglia. *Tissue Cell* **8**, 395–410 (1976).
204. Horridge, G. A. & Chapman, R. A. Sheaths of the motor axons of the crab Carcinus. *J. Cell Sci.* **S3-105**, 175–181 (1964).
205. Meyer-Rochow, V. B., Stephan, H. & Moro, S. D. Morphological and anatomical observations on the hairy eyes of males and females of the marine amphipoddulichia porrecta(crustacea, amphipoda, podoceridae). *Boll. Zool.* **58**, 59–69 (1991).
206. Lane, N. J. & Campiglia, S. S. The lack of a structured blood-brain barrier in the onychophoran Peripatus acacioi. *J. Neurocytol.* **16**, 93–104 (1987).
207. Lutaud, G., Woollacott, R. M. & Zimmer, R. L. The bryozoan nervous system. *Biology of bryozoans* **377**, 410 (1977).
208. Zimmer, R. L. & Woollacott, R. M. Anatomy of the larva of Amathia vidovici (Bryozoa: Ctenostomata) and phylogenetic significance of the vesiculariform larva. *J. Morphol.* **215**, 1–29 (1993).
209. Gruhl, A. & Bartolomaeus, T. Ganglion ultrastructure in phylactolaemate Bryozoa: evidence for a neuroepithelium. *J. Morphol.* **269**, 594–603 (2008).
210. Quiroga, S. Y. *et al.* Evolution of flatworm central nervous systems: Insights from polyclads. *Genet. Mol. Biol.* **38**, 233–248 (2015).
211. Biserova, N. M., Gordeev, I. I., Korneva, J. V. & Salnikova, M. M. Structure of the glial cells in the nervous system of parasitic and free-living flatworms. *Biol. Bull. Russ. Acad. Sci.* **37**, 277–287 (2010).
212. Rothe, B. H., Schmidt-Rhaesa, A. & Kieneke, A. The nervous system of Neodasys chaetonotoideus (Gastrotricha: Neodasys) revealed by combining confocal laserscanning and transmission electron microscopy: evolutionary comparison of neuroanatomy within the Gastrotricha and basal Protostomia. *Zoomorphology* **130**, 51–84 (2011).
213. Teuchert, G. The ultrastructure of the marine gastrotrichTurbanella cornuta Remane (Macrodasyoidea) and its functional and phylogenetical importance. *Zoomorphologie* **88**, 189–246 (1977).
214. Fernández, I., Pardos, F., Benito, J. & Roldán, C. Ultrastructural observations on the phoronid nervous system. *J. Morphol.* **230**, 265–281 (1996).
215. Temereva, E. N. & Tsitrin, E. B. Development and organization of the larval nervous system in Phoronopsis harmeri: new insights into phoronid phylogeny. *Front. Zool.* **11**, 3 (2014).
216. Kuzmina, T. & Temereva, E. Ultrastructure of ganglia in the brachiopod*Coptothyris grayi*and its phylogenetic significance. *J. Zoolog. Syst. Evol. Res.* **59**, 376–386 (2021).
217. Beckers, P., Krämer, D. & Bartolomaeus, T. The nervous systems of Hoplonemertea (Nemertea). *Zoomorphology* **137**, 473–500 (2018).
218. Le Marrec-Croq, F., Drago, F., Vizioli, J., Sautière, P.-E. & Lefebvre, C. The leech nervous system: a valuable model to study the microglia involvement in regenerative processes. *Clin. Dev. Immunol.* **2013**, 274019 (2013).
219. Turola, E., Furlan, R., Bianco, F., Matteoli, M. & Verderio, C. Microglial microvesicle secretion and intercellular signaling. *Front. Physiol.* **3**, 149 (2012).
220. Schmidt, J., Prinz, P. & Deitmer, J. W. Glial hyperpolarization upon nerve root stimulation in the leech Hirudo medicinalis. *Glia* **27**, 32–38 (1999).
221. Deitmer, J., Lohr, C., Britz, F. C. & Schmidt, J. Glial signalling in response to neuronal activity in the leech central nervous system. *Prog. Brain Res.* **132**, 215–226 (2001).
222. Deitmer, J. W. & Kristan, W. B., Jr. Glial responses during evoked behaviors in the leech. *Glia* **26**, 186–189 (1999).
223. Lohr, C. & Deitmer, J. W. Calcium signaling in invertebrate glial cells. *Glia* **54**, 642–649 (2006).
224. Hermans, C. O. Fine structure of the segmental ocelli of Armandia brevis (Polychaeta: Opheliidae). *Z. Zellforsch. Mikrosk. Anat.* **96**, 361–371 (1969).
225. Purschke, G. Structure of the prostomial appendages and the central nervous system in the Protodrilida (Polychaeta). *Zoomorphology* **113**, 1–20 (1993).
226. Baskin, D. G. Fine structure, functional organization and supportive role of neuroglia in Nereis. *Tissue Cell* **3**, 579–587 (1971).
227. Paemen, L. R. *et al.* Glial localization of interleukin-1 α in invertebrate ganglia. *Cell. Mol. Neurobiol.* **12**, 463–472 (1992).
228. Beckers, P. *et al.* The central nervous system of Oweniidae (Annelida) and its implications for the structure of the ancestral annelid brain. *Front. Zool.* **16**, 6 (2019).
229. Beckers, P., Helm, C. & Bartolomaeus, T. The anatomy and development of the nervous system in Magelonidae (Annelida) - insights into the evolution of the annelid brain. *BMC Evol. Biol.* **19**, 173 (2019).

230. Heuer, C. M., Müller, C. H., Todt, C. & Loesel, R. Comparative neuroanatomy suggests repeated reduction of neuroarchitectural complexity in Annelida. *Front. Zool.* **7**, 13 (2010).
231. Rice, M. E.
232. Elekes, K. Autoradiographic localization of monoamine uptake in the central nervous system of a marine mollusc (Mactra stultorum L., pelecypoda). *Neuroscience* **3**, 49–58 (1978).
233. Dos Santos, P. C., Gehlen, G., Faccioni-Heuser, M. C. & Achaval, M. Detection of glial fibrillary acidic protein (GFAP) and vimentin (Vim) by immunoelectron microscopy of the glial cells in the central nervous system of the snail *Megalobulimus abbreviatus*: GFAP and Vim in glial cells of Megalobulimus. *Acta Zool.* **86**, 135–144 (2005).
234. Gommerat, I. & Gola, M. Satellite glial cell responses to neuronal firing in the nervous system of Helix pomatia. *J. Membr. Biol.* **138**, 209–219 (1994).
235. Pentreath, V. W. Metabolic interactions between neurons and glial cells in leech and snail ganglia. in *Neuron—Glia Interrelations During Phylogeny* 161–196 (Humana Press, Totowa, NJ, 1995). doi:10.1007/978-1-59259-468-9_7.
236. Lockhart, S. T., Levitan, I. B. & Pikielny, C. W. Ag, a novel protein secreted from Aplysia glia. *J. Neurobiol.* **29**, 35–48 (1996).
237. López, J. C. Crystal-clear glia–neuron interactions: Glia. *Nat. Rev. Neurosci.* **2**, 380–380 (2001).
238. Lenhossék, M. v. Histologische Untersuchungen am Sehlappen der Cephalopoden: Hierzu 3 Figuren im Text und Tafel VI, VII u. VIII. *Arch. Mikrosk. Anat. (1865)* **47**, 45–120 (1896).
239. y Cajal, S. R. *Contribución Al Conocimiento de La Retina Y Centros ópticos de Los Cefalópodos*. (Unión Internacional de Ciencias Biológicas, Comité Español, 1930).
240. Barber, V. C. & Graziadei, P. The fine structure of cephalopod blood vessels. I. Some smaller peripheral vessels. *Z. Zellforsch. Mikrosk. Anat.* **66**, 765–781 (1965).
241. Young, J. Z. The retina of cephalopods and its degeneration after optic nerve section. *Philos. Trans. R. Soc. Lond. B Biol. Sci.* **245**, 1–18 (1962).
242. Stephens, P. R. & Young, J. Z. The glio-vascular system of cephalopods. *Philos. Trans. R. Soc. Lond. B Biol. Sci.* **255**, 1–12 (1969).
243. Young, J. Z. Neurovenous tissues in cephalopods. *Philos. Trans. R. Soc. Lond. B Biol. Sci.* **257**, 309–321 (1970).
244. Young, J. Z. The central nervous system of Loligo. I. The optic lobe. *Philos. Trans. R. Soc. Lond. B Biol. Sci.* **267**, 263–302 (1974).
245. Young, J. Z. The concept of neuroglia. *Ann. N. Y. Acad. Sci.* **633**, 1–18 (1991).
246. Gray, E. G. Electron microscopy of the glio-vascular organization of the brain of octopus. *Philos. Trans. R. Soc. Lond.* **255**, 13–32 (1969).
247. Bundgaard, M. & Abbott, N. J. All vertebrates started out with a glial blood-brain barrier 4-500 million years ago. *Glia* **56**, 699–708 (2008).
248. Bundgaard, M. & Abbott, N. J. Fine structure of the blood-brain interface in the cuttlefish Sepia officinalis (Mollusca, Cephalopoda). *J. Neurocytol.* **21**, 260–275 (1992).
249. Bogoraze, D. & Cazal, P. Recherches histologiques sur filesysteme nerveux du poulpe. *Arch. Zool. exp. gen* **83**, 413–444 (1944).
250. Case, N. M., Gray, E. G. & Young, J. Z. Ultrastructure and synaptic relations in the optic lobe of the brain of Eledone and Octopus. *J. Ultrastruct. Res.* **39**, 115–123 (1972).
251. Koenig, K. M., Sun, P., Meyer, E. & Gross, J. M. Eye development and photoreceptor differentiation in the cephalopod Doryteuthis pealeii. *Development* **143**, 3168–3181 (2016).
