# Supplementary material for: A single-cell atlas of the bobtail squid visual and nervous system highlights molecular principles of convergent evolution
Source: Nat Ecol Evol. 2025 Jun 6;9(7):1245–62. doi: 10.1038/s41559-025-02720-9 (PMC12240821; doi:10.1038/s41559-025-02720-9)
Supplement: Supplementary file 2 — Reporting Summary [file 41559_2025_2720_MOESM2_ESM.pdf]

nature portfolio

Corresponding author(s): Daria Gavriouchkina; Ferdinand Marlétaz, Daniel Rokhsar

Last updated by author(s): Feb 26, 2025

Reporting Summary

Nature Portfolio wishes to improve the reproducibility of the work that we publish. This form provides structure for consistency and transparency in reporting. For further information on Nature Portfolio policies, see our Editorial Policies and the Editorial Policy Checklist.

Statistics

For all statistical analyses, confirm that the following items are present in the figure legend, table legend, main text, or Methods section.

n/a	Confirmed	
☐	☒	The exact sample size (*n*) for each experimental group/condition, given as a discrete number and unit of measurement
☐	☒	A statement on whether measurements were taken from distinct samples or whether the same sample was measured repeatedly
☐	☒	The statistical test(s) used AND whether they are one- or two-sided<br>*Only common tests should be described solely by name; describe more complex techniques in the Methods section.*
☒	☐	A description of all covariates tested
☐	☒	A description of any assumptions or corrections, such as tests of normality and adjustment for multiple comparisons
☐	☒	A full description of the statistical parameters including central tendency (e.g. means) or other basic estimates (e.g. regression coefficient) AND variation (e.g. standard deviation) or associated estimates of uncertainty (e.g. confidence intervals)
☐	☒	For null hypothesis testing, the test statistic (e.g. *F*, *t*, *r*) with confidence intervals, effect sizes, degrees of freedom and *P* value noted<br>*Give P values as exact values whenever suitable.*
☒	☐	For Bayesian analysis, information on the choice of priors and Markov chain Monte Carlo settings
☒	☐	For hierarchical and complex designs, identification of the appropriate level for tests and full reporting of outcomes
☐	☒	Estimates of effect sizes (e.g. Cohen's *d*, Pearson's *r*), indicating how they were calculated

Our web collection on statistics for biologists contains articles on many of the points above.

Software and code

Policy information about availability of computer code

Data collection	SRA-Toolkit v2.11.3
Data analysis	Jellyfish v.2.2.7, bwa mem v.0.7.17, wtdbg2 v.2.5, Racon v.1.3.2, Scaff10X, Merqury v.1.1, minimap2 v.2.16, IsoSeq-3.0, BUSCO v.3.1.0, HiRise, Juicer v.e0d1bb7, Juicebox v.1.11.08, Star v.2.5.2,bcl2fastq (v2.19), Stringtie v1.3.3b, GMAP version 2019-02-26, Mikado v1.2.1, Taco, Portcullis v1.0.2, TransDecoder, Augustus v.3.3.3, RepeatModeler v.1.0.11, RepeatMasker v.4.0.7, PASA v.2.41, PfamScan v.1.6, Orthofinder v2.33,cellranger mkfastq v.3.1.0, UTR_extension_GTF.py v.1, R version 4.1.2, Seurat v.4.1.0, Amira v.6.5, ParaView , Fiji v.1.2.30/1.53, SAMtools v.1.9, BEDtools v2.25.0, Python 3.8.10,FigTree v.1.4.4,RAxML v.8.2.11.9, Adobe Photoshop 2021 22.4.3. release, Adobe Illustrator 2021 25.4.1 release

For manuscripts utilizing custom algorithms or software that are central to the research but not yet described in published literature, software must be made available to editors and reviewers. We strongly encourage code deposition in a community repository (e.g. GitHub). See the Nature Portfolio guidelines for submitting code & software for further information.

Data

Policy information about availability of data

All manuscripts must include a data availability statement. This statement should provide the following information, where applicable:

- Accession codes, unique identifiers, or web links for publicly available datasets
- A description of any restrictions on data availability
- For clinical datasets or third party data, please ensure that the statement adheres to our policy

Accession code and unique identifiers to previously publicly available datasets are listed in Methods. All sequence data associated with this project are available at the European Nucleotide Archive (project PRJEB52690) and Gene Expression Omnibus (accession number GSE203527).

Research involving human participants, their data, or biological material

Policy information about studies with human participants or human data. See also policy information about sex, gender (identity/presentation), and sexual orientation and race, ethnicity and racism.

Reporting on sex and gender	*Use the terms sex (biological attribute) and gender (shaped by social and cultural circumstances) carefully in order to avoid confusing both terms. Indicate if findings apply to only one sex or gender; describe whether sex and gender were considered in study design; whether sex and/or gender was determined based on self-reporting or assigned and methods used. Provide in the source data disaggregated sex and gender data, where this information has been collected, and if consent has been obtained for sharing of individual-level data; provide overall numbers in this Reporting Summary. Please state if this information has not been collected. Report sex- and gender-based analyses where performed, justify reasons for lack of sex- and gender-based analysis.*
Reporting on race, ethnicity, or other socially relevant groupings	*Please specify the socially constructed or socially relevant categorization variable(s) used in your manuscript and explain why they were used. Please note that such variables should not be used as proxies for other socially constructed/relevant variables (for example, race or ethnicity should not be used as a proxy for socioeconomic status). Provide clear definitions of the relevant terms used, how they were provided (by the participants/respondents, the researchers, or third parties), and the method(s) used to classify people into the different categories (e.g. self-report, census or administrative data, social media data, etc.) Please provide details about how you controlled for confounding variables in your analyses.*
Population characteristics	*Describe the covariate-relevant population characteristics of the human research participants (e.g. age, genotypic information, past and current diagnosis and treatment categories). If you filled out the behavioural & social sciences study design questions and have nothing to add here, write "See above."*
Recruitment	*Describe how participants were recruited. Outline any potential self-selection bias or other biases that may be present and how these are likely to impact results.*
Ethics oversight	*Identify the organization(s) that approved the study protocol.*

Note that full information on the approval of the study protocol must also be provided in the manuscript.

Field-specific reporting

Please select the one below that is the best fit for your research. If you are not sure, read the appropriate sections before making your selection.

☒ Life sciences ☐ Behavioural & social sciences ☐ Ecological, evolutionary & environmental sciences

For a reference copy of the document with all sections, see nature.com/documents/nr-reporting-summary-flat.pdf

Life sciences study design

All studies must disclose on these points even when the disclosure is negative.

Sample size	Tissues were sampled and dissociations were carried out using tissues from a single individual at a time.
Data exclusions	No data was excluded.
Replication	scRNA-seq was performed in replicates (4, 5, 6 , 5 for mature OLs, hatchling OLs, retinas, non-optic lobe perioesophageal brain respectively)
Randomization	All animal collections were performed randomly to ensure genetic variability.
Blinding	All animal collections were allocated blindly to any of the replicates of study.

Reporting for specific materials, systems and methods

We require information from authors about some types of materials, experimental systems and methods used in many studies. Here, indicate whether each material, system or method listed is relevant to your study. If you are not sure if a list item applies to your research, read the appropriate section before selecting a response.

Materials & experimental systems

n/a	Involved in the study
☐	☒ Antibodies
☒	☐ Eukaryotic cell lines
☒	☐ Palaeontology and archaeology
☐	☒ Animals and other organisms
☒	☐ Clinical data
☒	☐ Dual use research of concern
☒	☐ Plants

Methods

n/a	Involved in the study
☒	☐ ChIP-seq
☒	☐ Flow cytometry
☒	☐ MRI-based neuroimaging

Antibodies

Antibodies used	Rb Anti-FMRF-amide (Ab15348) 1:1000; Alexa 568 goat anti-rabbit IgG 1:250; Anti-acetylated tubulin (ab24610) 1:100; Alexa 568 goat anti-mouse (1:250)
Validation	Antibody cross-reactivity against Eberryi was predicted based on multiple sequence alignments (MSA) of targeted antigens with closely phylogenetically related species.Antibodies were then validated in immunohistochemistry with our own species.

Animals and other research organisms

Policy information about studies involving animals; ARRIVE guidelines recommended for reporting animal research, and Sex and Gender in Research

Laboratory animals	Euprymna berryi. We kept animals caught in the wild from both sexes and and all ages. Female individuals carrying eggs were permitted to lay eggs and individuals were reared to adulthood.
Wild animals	Adult Euprymna berryi of unknown ages were collected from the coast of Mie prefecture in Japan and transported to the lab. Animals were euthanized in the course of study using 4% ethanol in sterile-filtered natural seawater.
Reporting on sex	Genome was obtained utilising sperm from male individual. Due to unequal male:female ratio, male specimens were utilised for scRNA-seq, bulk RNA-seq, to preserve the few female individuals in culture. Sex of individuals at embryonic stages cannot be determined as they lack hectocotylus at this stage and is therefore unknown.
Field-collected samples	Adult Euprymna berryi of unknown age were collected from the coast of Mie prefecture in Japan and transported to Okinawa where they were acclimated to temperature (20°C) and pH (8.3) of a closed aquarium system in filtered natural seawater obtained from the shores of Okinawa, Japan (OIST Seragaki Marine Science Station). Animals were maintained essentially as described previously until they were sacrificed for experiments. Animals were exposed to a static 12:12 hour light:dark cycle. Tanks that housed the animals contained an enriched environment including natural substrate (autoclaved sand or crushed coral), parts of clay pots and natural rocks as dens. Mature animals were fed daily with Opossum shrimp or mysids, whereas Neomysis Japonica proved a suitable prey for hatchlings. Fresh glass shrimp, Palaemonetes spp. and frozen shrimp which were purchased in local grocery stores were fed to late juveniles and adults. Tanks were cleaned daily to remove uneaten food and waste matter. Prior to experiments, animals were euthanized using 4% ethanol in sterile-filtered natural seawater. Animals were allowed to breed freely. Hatchlings were obtained either from eggs provided by females impregnated in the wild or by breeding wild animals in the laboratory.
Ethics oversight	This study was carried out in accordance with procedures authorised by Guidelines for Proper Conduct of Animal Experiments by the Science Council of Japan. Despite the absence of legislation pertaining specifically to cephalopods in Japan, we aspired to abide by the highest standards in the field. All conducted experiments were therefore also in line with EU Directive 2010/63/EU and with the guidelines and the principles detailed in Anrews et al., 2013, Smith et al., 2013, Fiorit et al., 2015 and Dicristina et al., 2015. All experiments were approved by the Okinawa Institute of Science and Technology Graduate University Animal Care and Use Committee (approval ID: 2018-204). No transgenic animals were used in this study.

Note that full information on the approval of the study protocol must also be provided in the manuscript.

Plants

Seed stocks	*Report on the source of all seed stocks or other plant material used. If applicable, state the seed stock centre and catalogue number. If plant specimens were collected from the field, describe the collection location, date and sampling procedures.*
Novel plant genotypes	*Describe the methods by which all novel plant genotypes were produced. This includes those generated by transgenic approaches, gene editing, chemical/radiation-based mutagenesis and hybridization. For transgenic lines, describe the transformation method, the number of independent lines analyzed and the generation upon which experiments were performed. For gene-edited lines, describe the editor used, the endogenous sequence targeted for editing, the targeting guide RNA sequence (if applicable) and how the editor was applied.*
Authentication	*Describe any authentication procedures for each seed stock used or novel genotype generated. Describe any experiments used to assess the effect of a mutation and, where applicable, how potential secondary effects (e.g. second site T-DNA insertions, mosiacism, off-target gene editing) were examined.*
